# Syncopation as structure bootstrapping: the role of asymmetry in rhythm and language

**DOI:** 10.3389/fpsyg.2024.1304485

**Published:** 2024-02-15

**Authors:** Gaetano Fiorin, Denis Delfitto

**Affiliations:** ^1^Department of Humanities, University of Trieste, Trieste, Italy; ^2^Department of Cultures and Civilizations, University of Verona, Verona, Italy

**Keywords:** rhythm, meter, syncopation, syntax, hierarchy, linearization, bootstrapping

## Abstract

Syncopation – the occurrence of a musical event on a metrically weak position preceding a rest on a metrically strong position – represents an important challenge in the study of the mapping between rhythm and meter. In this contribution, we present the hypothesis that syncopation is an effective strategy to elicit the bootstrapping of a multi-layered, hierarchically organized metric structure from a linear rhythmic surface. The hypothesis is inspired by a parallel with the problem of linearization in natural language syntax, which is the problem of how hierarchically organized phrase-structure markers are mapped onto linear sequences of words. The hypothesis has important consequences for the role of meter in music perception and cognition and, more particularly, for its role in the relationship between rhythm and bodily entrainment.

## 1 Introduction

Syncopation is generally defined as the occurrence of a musical event on a metrically weak position preceding a rest on a metrically strong position [Huron, 2006; grounded on seminal work by Longuet-Higgins and Lee (1984)]. It represents an especially complex challenge in the study of the mapping between rhythm, intended as the linear temporal sequence in which musical events occur (Toussaint, 2013), and meter, the underlying cognitive structure within which musical events are perceived and interpreted according to influential models of rhythm perception such as the Generative Theory of Tonal Music (Lerdahl and Jackendoff, 1983), Dynamic Attending Theory (Large, 1994; Large and Kolen, 1994), and Predictive Coding (Friston, 2009). The challenge is made even more arduous by the observation that syncopation appears to be, among all forms of rhythmic complexity (Thul and Toussaint, 2008), the most effective in eliciting “the most desire to move and the most pleasure” (Witek et al., 2014, p. 1). Furthermore, we find syncopated rhythms among the most popular rhythms among different musical cultures and idioms (Toussaint, 2013). Syncopation is, thus, a powerful musical tool for making music that listeners can move to and find pleasure in listening to. The question we address here is why this is so – that is, what makes a deviation from temporal expectations such as syncopation so musically productive.

We first review some of the most prominent existing frameworks of meter (Section 2) and syncopation (Section 3) and consider how our research question is approached in other frameworks. We then suggest a novel hypothesis whereby syncopation is an especially effective strategy in eliciting the bootstrapping of hierarchically organized metric structures from linear sequences of musical events. Syncopated rhythms, by presenting an asymmetric distribution of musical events, are especially effective in suggesting a richer hierarchical organization in the underlying metric framework. We formulate this hypothesis by considering some recent empirical results and theoretical ideas concerning a parallel problem in natural language syntax: the problem of linearization. This is the problem of how hierarchically organized phrase-structure markers are mapped onto linear sequences of terminal nodes (Section 4). The hypothesis we propose has important consequences for the notion of musical meter and the role it plays in music perception and cognition, which we discuss in Section 5. Typically, meter is regarded as a governing framework, marking expectations concerning the temporal distribution of musical events. Conversely, our hypothesis suggests an approach whereby it is the linear form of rhythm that informs meter and the role of meter is that of offering a cyclic temporal framework of bodily entrainment, rather than perception.

## 2 Rhythm, meter, and rhythm-meter mapping

### 2.1 Rhythm and metric rhythm

In the most general sense, rhythm is “a patterned configuration of attacks that may or may not be constrained overall by a meter” (Powers, 2003, p. 723). In what follows, we will restrict our attention to rhythms that are constrained by a meter as it is only in the context of metric rhythm that syncopation occurs.

Minimally, a metric rhythm is a pulsation, a string of evenly spaced pulses, some of which are sounded (attacks) while other are silent (rests) (Toussaint, 2013, p. 5). The notations in (1) offer two different ways of representing a metric rhythm of eight pulsations of which the first, the second, the third and the fifth are attacks and all others are rests. The notation in (1a) represents the metric rhythm as a sequence of 1’s and 0’s; each number in the sequence corresponds to a pulse; a pulse marked by the number 1 is an attack; a pulse marked by the number 0 is a rest. The notation in (1b) (Breslauer, 1988) represents the same metric rhythm based on its durational patterns, that is, by listing the distance in number of pulses between (the onsets of) successive attacks, also called inter-onset interval (IOI).

(1)a.11101000

b.[1-1-2-4]

This view of rhythm obviously simplifies the reality of actual musical practice, where rhythms are produced by relying on a larger vocabulary of musical events than just attacks and rests. Modulations in amplitude, frequency, and timbre are powerful means in the hands of the instrumentalist to produce engaging musical rhythms. Still, as recognized since as early as Simon (1968), the listener’s ability to interpret the metric organization of a musical piece must also include the capacity to do so solely on the basis of the relative durations of IOI’s.

The restriction to metric rhythm also excludes a variety of phenomena that are nonetheless typically classified under the more general notion of rhythm. These range from Gregorian chants to melo-recitation in opera. As anticipated, the reason for this restriction is that syncopation, the topic of our study, only occurs in the context of metric rhythms.

### 2.2 Meter

According to the definition adopted, metric rhythm is characterized by an underlying pulsation – a sense of the temporal distance that regularly occurs between the pulses, irrespectively of whether these are materialized in the rhythm as rests or attacks (Honing, 2012). A common claim among musicologists is that the cyclic pulsation underlying metric rhythms encompasses a richer, possibly hierarchical, organization (Yeston, 1976; Lerdahl and Jackendoff, 1983; London, 2012) whereby some pulses are more salient (stronger) than others. This organization provides the listener with a relatively rich expectancy structure that assigns different degrees of expectation to different pulses (Jones, 1990; Large and Kolen, 1994; Ladinig et al., 2009; Rohrmeier and Koelsch, 2012).

While keeping in mind the limitations mentioned in the previous section, from this point on we will use the term “rhythm” to refer to the linear form of a metric rhythm. This specific notion of rhythm belongs to what Lerdahl and Jackendoff (1983, p. 48) refer to as “musical surface” and, for our purposes, can be described as a sequence of successive IOI’s.^1^ In contrast, we will use the term “meter” to refer to the, possibly hierarchical, organization of the underlying pulsation within which a rhythm unfolds.

Although the notion of meter finds broad support in the literature, the degree of complexity and rigidity of its inner organization remains debated. Lerdahl and Jackendoff (1983; see also Longuet-Higgins and Lee, 1984) propose a model of meter that is rigidly hierarchical and encompasses one or more intermediate levels between the pulsation and the downbeat. Yeston (1976) – a pioneer in applying the Schenkerian analytic methodology to the rhythmic domain – regarded meter as the result of the interaction between different “strata” of flat musical rhythms that are generally organized in a hierarchical fashion but can also stand in a more dynamic structural relationship with each other. A similar view is found in more recent models of meter such as those based on Dynamic Attending Theory (Jones, 1976). There is also discussion concerning the psychological reality of the inner structural organization of meter. It has been especially difficult to test intermediate levels of metric salience experimentally (Parncutt, 1994; Ladinig et al., 2009; Song et al., 2013; but see Katz et al., 2015) and sensible differences in sensitivity to intermediate metric levels have been observed when comparing trained musicians to non-musicians (Palmer and Krumhansl, 1990).

### 2.3 Rhythm-meter mapping

To be of explanatory value, the distinction between rhythm (intended as a cyclic, linear sequence of IOI’s) and meter (a hierarchically organized pulsation) must be supplemented with an explicit formulation of the principles that govern the mapping between the two. In the reality of musical practice, listeners infer the meter of a metric musical performance with no conscious effort or volition and based on a large variety of factors. These may range from properties internal to the musical performance (such as its amplitude envelops, its melodic and harmonic organization, and its timbral modulations) to culturally established metric templates characteristic of the music idiom of the performance at stake (Jones, 1990; London, 1990). This said, our focus in this contribution will be restricted, in the spirit of Simon (1968), to how the relative durations of the IOI’s of a metric rhythm relate to its underlying meter. In what follows, we will briefly review three of the most influential theoretical models of the mapping between meter and rhythm.

#### 2.3.1 Well-formedness and preference rules

**Figure 1:**
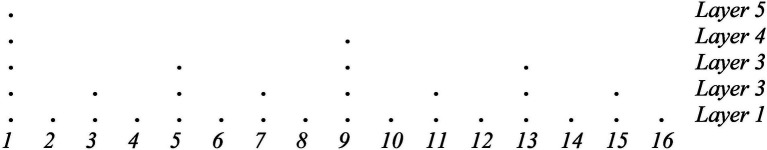
Analysis of a canonical 4/4 meter in western tonal music in the style of Lerdahl and Jackendoff’s (1983). GTTM, Generative Theory of Tonal Music.

Figure 1 offers an analysis of a canonical 4/4 meter in western tonal music in the style of Lerdahl and Jackendoff’s (1983) Generative Theory of Tonal Music (GTTM). The dot-notation in the figure comprises five hierarchically organized layers of metric saliency. At the lowest level (Layer 1) we find the pulsation and at the highest level (Layer 5) the downbeat. The highest a level, the highest the saliency of its pulses and, accordingly, the expectation that an attack would occur in their position.

GTTM provides two sets of rules constraining, respectively, the well-formedness of a meter and the possible associations between rhythm and meter.

Below we present the meter well-formedness rules (MWFR) that are relevant to the present discussion. Compared to their original formulation, the rules presented below are slightly simplified in those aspects that are not directly relevant to our goals and some of the terms are adapted to the terminology we have adopted so far (for example, we use the term pulse whenever GTTM talks of beat).

(MWFR 1) Every attack point must be associated with a pulse at the smallest level of metrical structure.

(MWFR 2) Every pulse at a given level must also be a pulse at the smaller level.

(MWFR 3) At each metrical level, strong pulses must be spaced either two or three pulses apart.

(MWFR 4) Each metrical level must consist of equally spaced pulses.

MWFR 1 can be regarded as a quantization constraint, aligning the actual musical events (attacks and rests) to the pulses of the meter. MWFR 2 is a constraint on the hierarchical organization of the levels in the meter. MWFR 3 and 4 are constraints on IOI’s between pulses belonging to the same level.^2^ These last rules are idiom specific and explicitly conceived for classical western tonal music and its preference for isochronous (binary or ternary) meters. The possibility is left open that different musical idioms may differ in this respect.

Rules of the second group are called meter preference rules (MPR). GTTM offers a number of such rules, also related to how factors such as stress, pitch, harmony and length are mapped to metrical structure. Here, we will focus only on those rules that are relevant to the present discussion.

(MPR 1) When two or more groups or parts of groups can be constructed as parallel, they preferably receive parallel metrical structure.

(MPR 3) Prefer a metrical structure in which attack onsets coincide with strong pulses.

**Figure 2:**
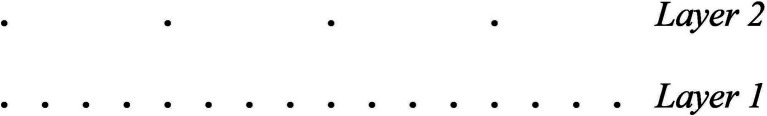
Metric analysis predicted for the rhythm 1110111011101110 by MPR1.

MPR 1 relies on the notion of group, which is central to the GTTM’s framework. The rule captures the intuition that a rhythm such as (2) is preferably interpreted within a metrical structure that repeats every four pulses, such as the one in Figure 2.

(2)1110111011101110

**Figure 3:**
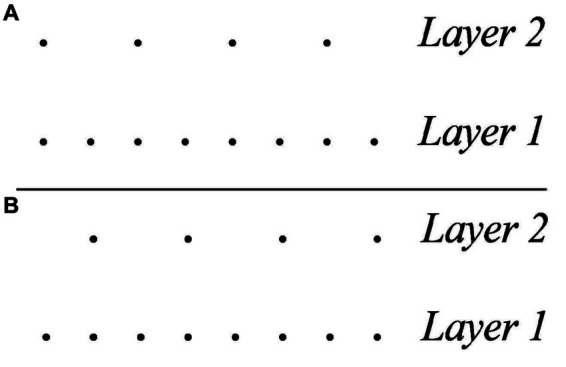
**(A)** Preferred metrical analysis of the rhythm 10101010 according to MPR3. **(B)** Less preferable metrical analysis of the rhythm 10101010 according to MPR3.

MPR 3 expresses a preference for attacks to coincide with strong pulses and rests with weak pulses, so that, for example, it is preferable to interpret (3) within the metrical structure in Figure 3A, rather than that in Figure 3B.

(3)10101010

**Figure 4:**
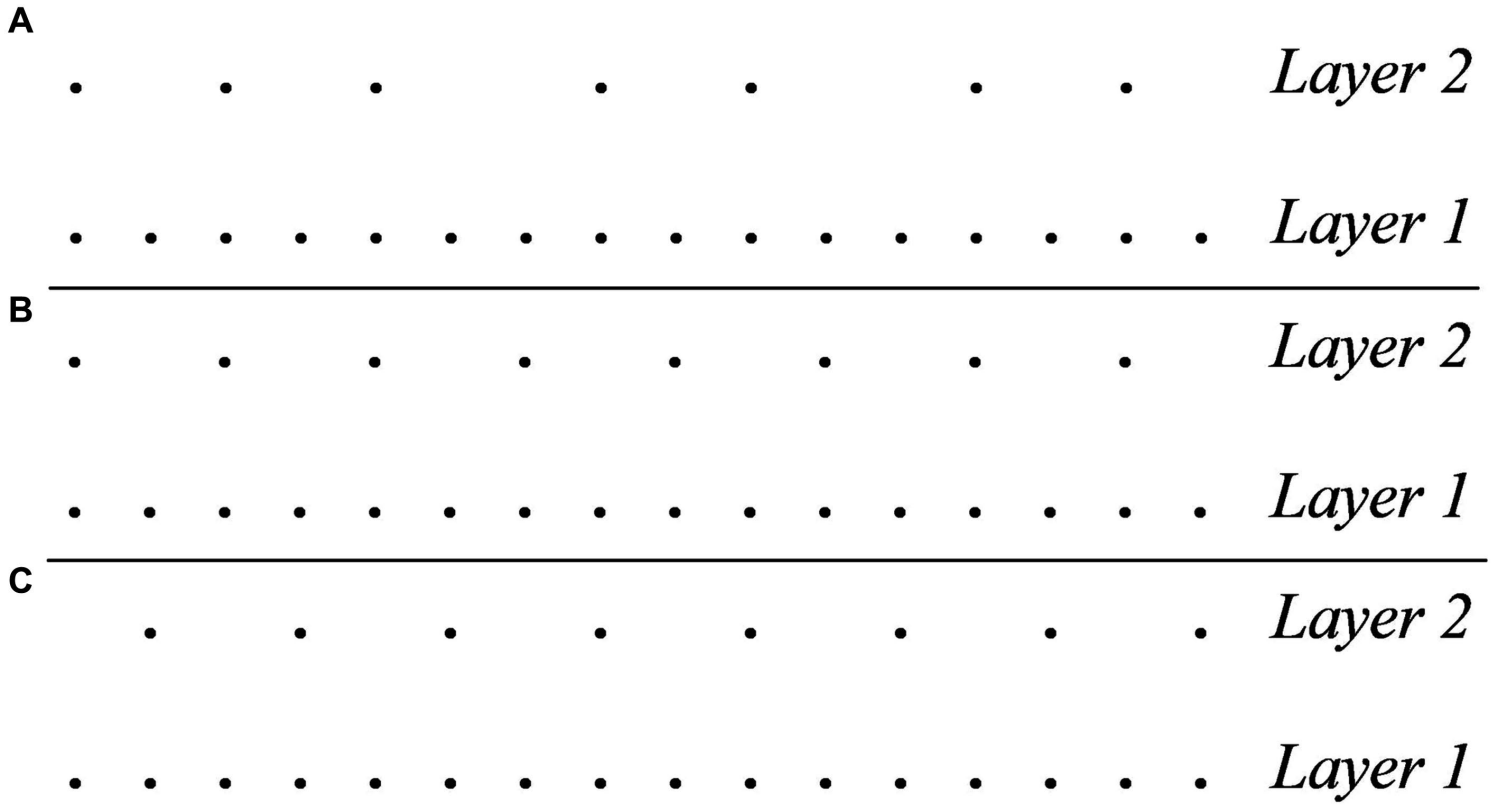
**(A)** Analysis of the rhythm 1010100101001010 that satisfies MPR 3 but violates MWFR 4. **(B)** Most optimal analysis of the rhythm 1010100101001010 that satisfies MWFR 4 but violates MPR 3. **(C)** Least optimal analysis of the rhythm 1010100101001010 that satisfies MWFR 4 but violates MPR 3.

An important aspect of the system of GTTM is that preference rules are not hard constraints whose violation implies some form of ungrammaticality. Some musical forms may be such that they cannot satisfy all the rules. In that case, the interpreter still relies on the rules to uncover the underlying meter by looking for the interpretation that satisfies the most rules and minimizes their violations. To see an example, the metric analysis of (4) in Figure 4A satisfies MPR 3 but violates MWFR 4. To satisfy MWFR 4 we must violate MPR 3. In this case, there are two possible analyses, reproduced in Figures 4B,C. Ultimately, the analysis in Figure 4B is preferable because it is the metric structure that produces less violations.

(4) 1010100101001010

The question arises of why there should exist rhythms, such as (4), that violate the preference rules. We will return to this question in Section 3.

#### 2.3.2 Dynamic attending theory

Dynamic Attending Theory (DAT) is a neurocognitive model of how temporal expectations are generated in real-time (Large and Kolen, 1994; McAuley, 1995; Large and Jones, 1999; Barnes and Jones, 2000; Jones, 2016). Central to it, is the notion of entrainment, intended, in the context of DAT, as a biological process of synchronization between external events and internal attending mechanisms. These mechanisms are assumed to have an oscillatory nature (arguably related to electrophysiological oscillations at the neural level), to have a tendency toward stability, but also to be able to adapt to expectancy violations. Another important assumption of DAT is that the attending system is able to entrain simultaneously to multiple oscillations of different temporal resolutions. Furthermore, when multiple oscillations occur together, they can resonate with each other and mutually reinforce when their peaks coincide. In this framework, meter emerges from the interaction between multiple attentional periodicities of different temporal resolutions.

To see an example, when listening to a rhythm of period *t* in what would be signed in western tonal notation as a 4/4 meter, the listener’s attention would be captured by periodicities of time *t* as well as periodicities of time *t*/2, *t*/4, *t*/8, and so on. The compound of these periodicities is a meter that reaches its maximal attention peak at *t*, a lower attention peak at *t*/2, an even lower attention peak at *t*/4, and so on, ultimately expressing an expectancy framework that is not dissimilar to that of the GTTM model (as expressed by the dot-notation in Figure 1).

In fact, the model of meter provided by DAT is not entirely incompatible with that of GTTM although there are some significant differences between the two models. GTTM focuses on the analytic properties of the final representation of meter and, therefore, does not provide a model of the actual cognitive mechanisms that support the construction of meter in real-time music perception or the real-time adaptation of a meter to a rhythm that violates it. In contrast, DAT shifts its focus to the bottom-up attentional processes that support the real-time bootstrapping of meter from the rhythmic surface and is also able to capture top-down processes such as the mechanisms of real-time detection of and adaptation to expectancy violations. Therefore, compared to GTTM, the DAT model offers a more flexible framework for explaining the dynamic interplay between rhythm and meter (Honing, 2012).

#### 2.3.3 Predictive coding

Predictive Coding (PC) is a theory of human (and, in some of its generalizations, mammal) neurocognition grounded on Helmholz’s insight that the brain is a predictive machine whose task is generating predictive models of forthcoming events and testing them against the information gathered by the senses [see Clark (2013) for an overview]. In its most recent incarnations (Friston, 2009), PC maintains that brain networks are organized hierarchically and that the flow of information between the lower and higher layers is bidirectional. The lower layers collect sensorial information and test it against the current internal model. The prediction error that is detected is then sent upward to the higher layers, which encode the prior probabilities of the model. Depending on the magnitude of the prediction error detected, the higher levels either confirm or correct the existing model. The resulting model is then sent downward to the lower layers to be tested against the sensorial input, generating a feedback loop whose goal is that of minimizing prediction error.

As a predictive system that is hierarchically organized and allows for information about prediction errors to flow bidirectionally, PC appears particularly well suited to explain the multi-layered predictive processes of meter perception: “For meter perception, PC can explain how lower levels, e.g., events at the eighth-note level, provide metric information about the whole-note level and the salience of the downbeat (feed forward). At the same time, the whole-note level, as marked by the most salient beat, the downbeat, provides a metric framework according to which the eighth-notes at the lower level are heard (feedback). This PC way of understanding metric hierarchies emphasizes the mutual relationship between bottom-up and top-down processes.” (Vuust and Witek, 2014, p. 5; see also Vuust et al., 2009, 2018; Vuust and Witek, 2014; Gebauer et al., 2015; Lumaca et al., 2019).

The PC model of meter has obvious commonalities with both the GTTM model and the DAT model, in particular, its reliance on a multi-layered, hierarchical organization of the predictions made at different temporal resolutions. Similarly to DAT, it differs from GTTM in that it offers a dynamic model of both the bottom-up processes that support the bootstrapping of meter in real-time music perception and the top-down process of real-time adaptation to expectancy violations.

#### 2.3.4 Linearization, antisymmetry, and structure bootstrapping in natural language syntax

**Figure 5:**
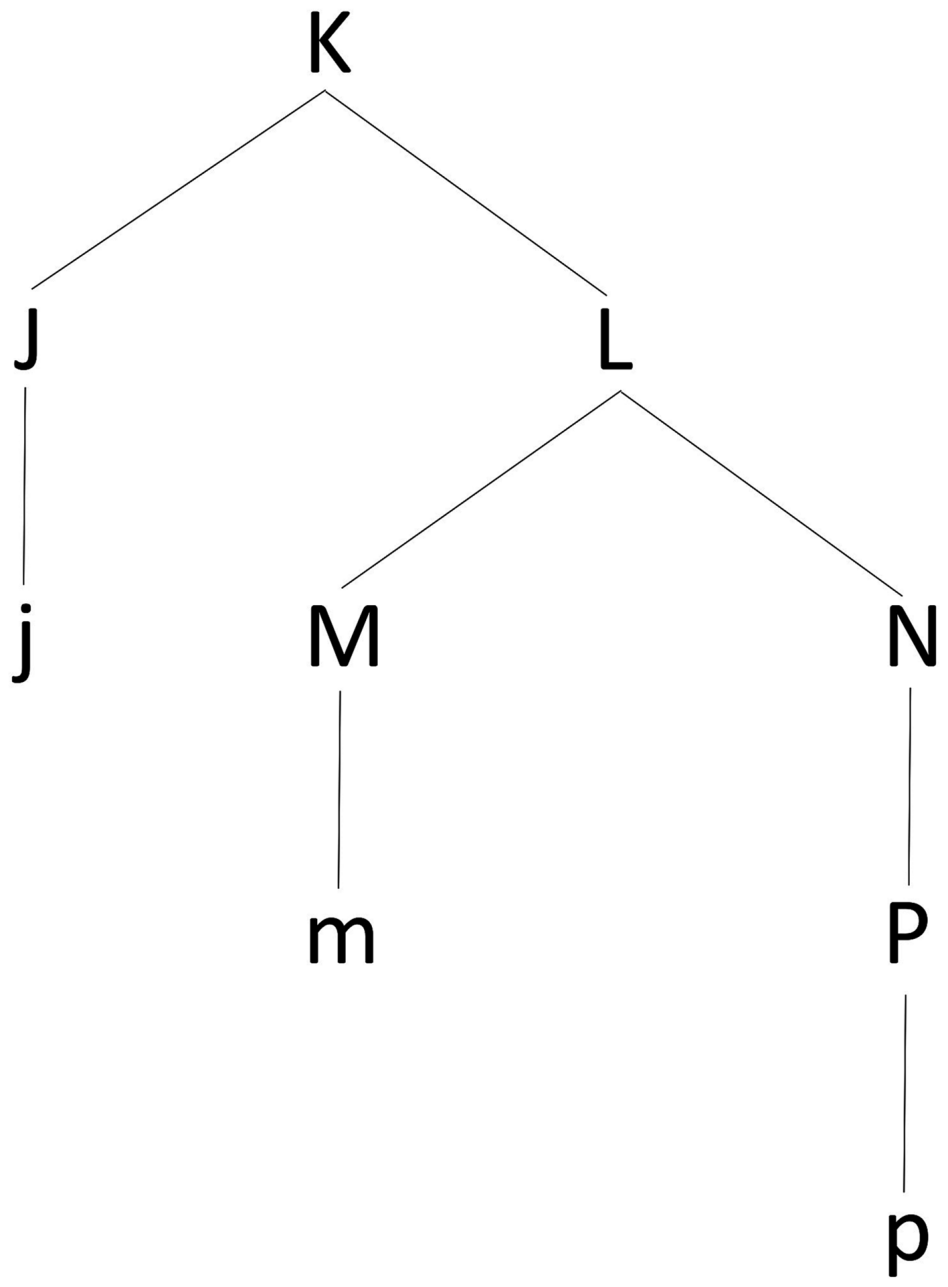
An abstract representation of a phrase-structure marker in the form of a syntactic tree. The tree has terminal nodes j, m, and p. These corresponds to the phonological exponents (terminals) of the tree structure, that is, the elements (typically words) that must be spelled-out. Conversely, the capital letters corresponds to categorial projections (non-terminals, or ‘invisible’ nodes) of these terminal nodes, expressing either their syntactic label (for example, J would be the syntactic label of j; a determiner such as “the,” for example, would be labeled as D, meaning “Determiner”) or the syntactic category resulting from the combination of two nodes (for example, a combination of a determiner and a noun, would be labeled as DP, meaning “Determiner Phrase”). According to the LCA, the linear order of the terminal nodes in a syntactic structure is read off inferred from the syntactic structure itself by translating all relations of asymmetric c-command between the nodes in the tree into relations of linear order (most typically, precedence). Applied to the phrase-structure in the figure, the LCA provides the following result. The pairs of non-terminals in a relation of asymmetric c-command are the following: <J, M>, <J, N>, <J, P>, <M, P>. This induces the following relations of precedence on the corresponding terminals: j<m, j<n, j<p, and m<p. In turn, these relations determine the string /jmp/.

The problem of understanding the mapping between rhythm –a linear sequence of musical events – and meter – an abstract, hierarchically organized framework for the interpretation of rhythm – finds a parallel in the domain of natural language syntax with the problem of linearization – how, that is, a two-dimensional phrase-structure marker such as the one in Figure 5 is mapped into a spellable linear order. In Figure 5, the terminal nodes j, m, and p stand in a hierarchical relation of dominance. The problem of linearization concerns how these terminal nodes are ordered according to a relation of precedence/subsequence so that they can be spelled out as a linear sequence.

The most influential hypothesis addressing this issue in the tradition of generative linguistics is Kayne’s (1994) theory of Antisymmetry and its Linear Correspondence Axiom (LCA).

Given a tree-diagram (phrase-marker), be X the set of non-terminals and d(X) the corresponding set of terminals (the members of d(X) will be the terminals dominated by each member of X; d(X) will be the image of X under d). Be now A the set of non-terminals of a particular phrase-marker such that A contains all the pairs <X_j_,Y_j_> where X_j_ asymmetrically c-commands Y_j_ (X_j_ commands Y_j_ but Y_j_ does not c-command X_j_); the image under d of A will be the set d(A) of ordered pairs <a_j_,b_j_> such that a_j_ belongs to d(X_j_) and b_j_ belongs to d(Y_j_): {<a_j_,b_j_>}. LCA is then defined as below:

(LCA) d(A) is a linear ordering of T (T = the set of terminals)

(DOMINANCE) Node α dominates node β iff α is in a higher position in the tree than β such that you can trace a line from α to β going only downwards.

(IMMEDIATE DOMINANCE) Node α immediately dominates node β iff α dominates β and there is no node γ such γ dominates β and α dominates γ.

(SISTERHOOD) Two nodes α and β are sisters if there is a node γ (their mother) which immediately dominates both α and β.

(C-COMMAND) α c-commands β iff

- α and β are categories
- no segment of α dominates β
- every category that dominates α dominates β (May, 1985)

(ASYMMETRIC C-COMMAND) α asymmetrically c-commands β iff

- α c-commands β and
- β does not c-command α

Given (LCA), Antisymmetry corresponds to the requirement that the ordering of the set of terminals T in a phrase-marker P be induced by the pairwise ordering d(A). The crucial insight is that <a,b> belongs to d(A) (terminal a is ordered with respect to terminal b) if and only if the non-terminal dominating a asymmetrically c-commands the non-terminal dominating b. In this way, linear ordering is made dependent on the satisfaction of a specific structural requirement. Applied to the phrase-structure marker in Figure 5, LCA predicts the linear order j < m < p (where < symbolizes linear precedence), given that J asymmetrically c-commands both M and P and that M asymmetrically c-commands P.

Antisymmetry has a number of important consequences, such as predicting, under some additional assumptions, a restricted number of possible world orders across world languages, and has undergone a number of refinements since its original formulation (see especially Moro, 2000).

One important aspect of approaches to linearization such as Kayne’s LCA and its derivates is that they are top-down in the sense that they apply their algorithms to already existing syntactic structures in order to provide corresponding linear orders. These approaches are in line with the general wisdom that linear order does not concern syntactic structures themselves but is an interface issue, which comes into play only at the point when abstract syntactic structures must be translated into spell-out instructions that are compatible with the physiological properties of the articulatory-perceptual system, in particular the fact that we humans can only pronounce one word at a time (there are different declinations of this view, applying with different degrees of strength; for an overview see Kayne, 2022).

An alternative to this approach is the Bootstrapping Principle (BP)^3^ by Vender et al. (2023) and Delfitto (2023). This route from order to structure capitalizes on the main insight of so-called neo-constructionist approaches, according to which “we might characterize the bare bones of syntax in terms of the hierarchical arrangement of morphemes, the minimal building blocks of grammar” (Marantz, 2013), and more particularly on the existence of a Universal Extended Projection (UEP) of lexical categories in syntax (Grimshaw, J. 1991. Extended Projection. Ms, Brandeis University, Waltham, MA and much subsequent literature). To exemplify, consider the sequence *seqN*, consisting of all functional elements in the extended projection of a nominal root (as for instance the sequence *Determiner-Number-Gender-Adjective-n*). Each element in the sequence selects (or is selected) by the adjacent elements (for instance, *Number* selects *Gender*, and *n* is selected by *Adjective*). Intuitively, if x_j_ selects x_i_, we want x_j_ to project (for instance, if *Number* selects *Gender* within *seqN*, we want *Number* to project): x_j_ is a new (invisible) node branching into x_i_ and x_j_. This result is achieved by applying bootstrapping as defined in (BP) below, which ensures the transition from a linear array of symbols to a hierarchically organized array of symbols). More precisely, if selection has been interpreted as *precedence* (the selectee precedes the selector, giving rise to the sequence *n-Adjective-Gender-Number-Determiner*), it follows that the binary subsequence x_i_-x_j_ of *seqN* gets mapped into the constituent x_j_; whereas if selection has been interpreted as *subsequence* (the selector precedes the selectee, giving rise to the sequence *Determiner-Number-Gender-Adjective-n*), it follows that the substring x_j_-x_i_ should correspond to the constituent x_j_.

(BP)i.Given the chunk [α, β], if α precedes β, then α is contained in β (*precedence*)

ii.Given the chunk [α, β], if α precedes β, then α contains β (*subsequence*)

BP approaches linearization from the opposite perspective of Antisymmetry theory as it regards linear order as a constitutive feature for the construction of a hierarchical syntactic space. BP is in fact intended as the algorithm that maps linear order into a two-dimensional syntactic space based on the relation of dominance/containment. It provides a bottom-up bootstrapping algorithm in the sense that it builds relations of containment from relations of precedence/subsequence.^4^

BP is supported by behavioral experimental results obtained using an artificial grammar learning paradigm where subjects are tested with respect to their capacity to predict elements – within an abstract sequence of 0’s and 1’s produced by a Fibonacci-grammar – that cannot be predicted, demonstrably, by means of statistically-based linear computations (Vender et al., 2019, 2020, 2023). The results suggest that subjects’ anticipatory skills are based on the capacity of labeling constituents such as [01], in which 0 < 1, as 1’s, in agreement with (BP): if 0 < 1, then 1 must dominate 0, which is the case if it is 1, and not 0, that labels [01] (Vender et al., 2023).

## 3 Syncopation

Syncopation offers an especially interesting case-study for understanding the relationship between rhythm and meter because it is a case in which the rhythmic surface explicitly deviates from metric expectations.

There are two main facts about syncopation that, in our opinion, should be accounted for by a theory of meter and its mapping with rhythm. Firstly, although syncopation is one of many forms of rhythmic complexity (Longuet-Higgins and Lee, 1984; Fitch and Rosenfeld, 2007), when compared to the other measures of rhythmic complexity, it appears to be a most appropriate predictor of perceived rhythmic complexity; Thul and Toussaint (2008) “found that measures of syncopation outperformed other measures in explaining the behavioral data from four separate studies. The data comprised of judgments regarding perceptual, metric and performance complexity of rhythmic patterns. It was found that models of syncopation better explained the variability in these judgments, compared to for example standard deviation and entropy (i.e., the degree of uncertainty in a random sample, from an information theory perspective)” (Witek et al., 2014, p. 1). Furthermore, syncopation also appears to be the most “important structural factor in embodied and affective responses to groove” (Witek et al., 2014, p. 1).^5^ Syncopation is, in other words, what makes listeners experience the urge to move to the music and with other listeners and feel good. In particular, Witek et al. (2014, p. 1) report that “[w]hile entropy was found to be a poor predictor of wanting to move and pleasure, […] medium degrees of syncopation elicited the most desire to move and the most pleasure, particularly for participants who enjoy dancing to music.” In fact, the study reports an inverted U-shape relationship between the degree of syncopation of a rhythm and (ratings of) the urge to move and pleasure experienced when listening to the rhythm.^6^

The second, potentially related factor, is that syncopated rhythms enjoy extensive popularity across different musical idioms and cultures (Toussaint, 2013; Witek et al., 2020). The rhythm in (5), most commonly known by its Cuban name *tresillo*, offers a paradigmatic example (Floyd, 1999; Acquista, 2009; Biamonte, 2018; Jajoria et al., 2021).

(5)a.10010010

b.[3-3-2]

The tresillo has played an important role in research on musical rhythm, both in ethnography and musical theorizing, as is found in a large variety of musical cultures: “The durational patter [3–3-2], popular in Central Africa, is most famously known as the Cuban tresillo (*tres* in Spanish means three), and is widely used in the circum-Carribean. However, it forms part of almost every music tradition throughout the world, and dates back to at least thirteenth-century Baghdad, where it was called al-thakil al-thani. It is a traditional rhythm played on the banjo in bluegrass music. It was used extensively in the American rockabilly music of the 1950’s for bass, saxophone, or piano. It is sometimes referred to as the habanera rhythm or tumbao rhythm, although the term habanera usually refers to the four-onset rhythm [3-1-2-2], which is less syncopated than the tresillo since it inserts an additional attack in the middle of the cycle. The habanera rhythm is also known as the tuba francesa. If to the habanera a fifth onset is inserted at pulse five, then the resultant rhythm [3–1–1-1-2] is the bomb from Puerto Rico. On the other hand, if the third onset of the tresillo pattern is deleted, one obtains the prototypical Charleston rhythm [x.x….]” (Toussaint, 2013, p. 14–15; notice that in the quote the term onset is used to refer to what we have so far referred to as attack).

The tresillo is also at the basis of a number of other more complex rhythms such as the Bossa nova and samba rhythms. It is also the first-half of the 3:2 clave or the second of the 2:3 clave. It is also the most common rhythm shape of bass line in Latin music (called tumbao) although it is typically played while omitting the first attack. It is also a frequent rhythmic element of jazz, rock, R&B, and dance music. It is an Euclidean rhythm (Toussaint, 2005) as it represents the most even distribution of three attacks over eight pulses. It has an important counterpart in the rhythm called cinquillo (6), which is also a Euclidean rhythm representing the most even distribution of five attacks over eight pulses and which is specular to the tresillo.

(6)a.10110110

b.[2-1-2-1-2]

What is important to observe for the purposes of the present discussion is that the tresillo is commonly interpreted, in all of its various incarnations, in the context of a 4/4 meter, a fact already recognized by Jones (1959; see also Kolinski, 1973; Agawu, 2003). This means that the tresillo is a syncopated rhythm, given that its second attack occurs on a metrically weak pulse immediately preceding a rest on a metrically strong pulse.

Indeed, the tresillo is not the only syncopated rhythm. What is relevant to observe here is that one of the most popular rhythms among a vast spectrum of musical idioms is, in fact, a syncopated rhythm.

Taken together, these two observations call for a theory of syncopation that, on top of unfolding from an explanation of the structural relationship between rhythm and meter, also explains why syncopation is so effective in eliciting desire to move and pleasure and so popular across different musical cultures.

### 3.1 Theories of syncopation

#### 3.1.1 Syncopation in GTTM

**Figure 6:**
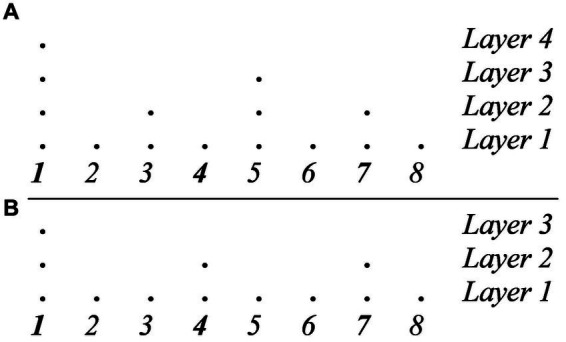
**(A)** Metric analysis of the tresillo rhythm that satisfies WFR 4 but violates both MPR 3 and MPR 1. **(B)** Metric analysis of the tresillo rhythm that satisfies MPR 1 and MPR 3 but violates WFR 4.

Syncopation poses a challenge to GTTM’s theory of meter. Consider again, as a paradigmatic example, the tresillo rhythm discussed above and compare the two metrical analyses in Figure 6. The analysis in Figure 6A produces a violation of MPR 3 (the second attack does not coincide with a strong pulse) as well as a violation of MPR 1 (the two parallel groups 100-100 do not receive parallel metrical structures). Conversely, the analysis in Figure 6B satisfies both MPR 1 and MPR 3 but produces a violation of MWFR 4 as now there is a metrical level that does not consists of equally spaced pulses. To be sure, both analyses are attested in actual musical practice. The meter in Figure 6B is that of a traditional Bulgarian rhythm (see, for example, Béla Bartók, Mikrokosmos, 153, Six dances in Bulgarian rhythm, n. 6). Yet, the most common metric analysis of the tresillo is the one in Figure 6A. The question, then, is why this is so and why a rhythm such as the tresillo is so popular and successful when it violates two MPR’s and is not trumped by a more optimal rhythm that does not violate them.

There is an intuition that violating the metric rules is a means to introduce tension and that doing so helps capturing and maintaining the listener’s attention (Spiech et al., 2022). This seems to be Lerdahl and Jackendoff’s view, for example, on the use of syncopation in Jazz: “The stylistic norm [of Jazz] is not simply stresses on weak beats; it consists rather of a number of strategies aimed at increasing local metrical tension. The normal preference rules do not fail to apply; in fact they are exploited as a means of creating the desired metrical tension, which results from a conflict among rules” (Lerdahl and Jackendoff, 1983, p. 279). According to Temperley (1999), the argument is implausible. In his study of syncopation in rock music, Temperely considers a number of melodies of popular rock songs featuring syncopation and observes that these melodies have very little sense of rhythmic instability or metric ambiguity, as opposed to other possible settings of the same melodies that would be more obviously compatible with the underlying meter (Temperley, 1999, p. 26). There is, in fact, some empirical evidence against the view that relates syncopation to metric tension. In a study by Keller and Schubert (2011), musicians rated pairs of unsyncopated and syncopated musical phrases for perceived complexity, enjoyment, happiness, arousal, and tension. The authors report that “overall, syncopated patterns were more enjoyed, and rated as happier, than unsyncopated patterns, while differences in perceived tension were unreliable. Complexity and arousal ratings were asymmetric by serial order, increasing when patterns moved from unsyncopated to syncopated, but not significantly changing when order was reversed. These results suggest that syncopation influences emotional valence (positively), and that while syncopated rhythms are objectively more complex than unsyncopated rhythms, this difference is more salient when complexity increases than when it decreases.” (p. 142).

In order to provide a more formal theory of the mapping between syncopated rhythmic surfaces and their underlying metric analysis within the framework of GTTMs, Temperley (1999) proposes an intermediate level of analysis between rhythm and meter. In GTTM, the musical surface of a piece represents the input of the metrical analysis:

MUSICAL SURFACE > METRICAL ANALYSIS

Temperley proposes that two levels must be distinguished within the musical surface: a ‘surface’ musical surface, henceforth simply referred to as surface structure, and a ‘deep’ musical surface, henceforth simply referred to as deep structure.

SURFACE STR > DEEP STR > METRICAL ANALYSIS

The deep structure differs from the surface structure in that all syncopated events are ‘de-syncopated’ according to the Syncopation Shifting Rule (SSR; we have adapted the rule, originally designed for melodies, to apply to rhythms)^7^:

(SSR)

In inferring the deep structure of a rhythm, any attack may be shifted forward by one beat at a low metrical level. The order of attacks in a line in the deep structure must be the same as their order in the surface structure.

According to this view, syncopated rhythms are surface deviations of rhythms that are not syncopated at a deeper level of representation. The idea that syncopated rhythms are the result of processes of transformation finds its roots in Schenkerian approaches to rhythm, such as Komar (1971) and Schachter (1980) and has recently found experimental and computational support in the work of Sioros and Guedes (2014).

The question remains, however, why syncopation should exist at all. Temperley (1999, p. 34–37) suggests three relevant observations: (a) syncopation allows for a variety of rhythmic surface structures from a single deep structure without endangering the stability of the underlying rhythm; (b) in the context of sung music, syncopation allows some degree of useful flexibility in fitting the melody to the rhythm of the lyrics; (c) syncopation may be exploited to suggest a latent meter that is different from the actual meter – for example, providing a three-over-two feeling – or anticipating a forthcoming change of meter – for example, from a duple to a triple meter.^8^

These observations are relevant in the context of syncopation in sung rock music but it is not straightforward that they extend to rhythmic syncopation more generally. To begin with, it is questionable whether Temperley’s model applies correctly to syncopation as it is featured, for example, in popular syncopated rhythms such as the tresillo. According to Temperley’s model, the surface structure of the tresillo should be the surface outcome of the deep structure in (7).

(7)a.10001010

b.[4-2-2]

This is questionable. The tresillo, as mentioned above, is a widely attested musical rhythm whose popularity extends across musical cultures and idioms. Conversely, the rhythm in (7), although a perfectly plausible and certainly attested rhythmic sequence (for example, as the second half of the cha-cha rhythm), does not enjoy the same wide-spread significance across musical cultures. This puts into question the idea that the tresillo is but a syncopated variation of an un-syncopated deep structure such as (7).

A further issue concerns the so-called habanera rhythm (8), a common variant of the tresillo which differs from it only in that it has an extra attack on pulse (8)

(8)a.10011010

b.[3-1-2-2]

The co-occurrence of an attack in pulse 4 (the syncopated position) and pulse 5 (the un-syncopated position) puts into question the idea that the syncopated attack of the tresillo is the result of the SSR rule. It is also true that the habanera has been analyzed, at least in some of its uses, as the combination of the tresillo with the so-called backbeat of the underlying 4/4 meter (Peñalosa, 2010, p. 42). This would support the view of syncopation as a strategy to enrich the surface structure of a rhythm while maintaining it grounded on a regular metric structure. We should notice, however, that it is not obvious that the notion of backbeat is entirely equivalent to that of a rhythm deep structure.

One aspect of the tresillo that confirms Temperley’s observations is its three-over-two feeling. As observed, the tresillo corresponds to the most even distribution of three attacks over eight pulses [in the terms of Toussaint (2005) it is a Euclidean rhythm] and can be regarded as a triplet quantized to fit a binary meter.^9^ The question remains, however, why a syncopated rhythm such as the tresillo should enjoy such popularity, as attested by its frequency across musical idioms and cultures, in contrast to its unsyncopated version. The question also remains why syncopated rhythms are so effective in eliciting bodily entrainment.

#### 3.1.2 Syncopation in DAT

From the perspective of DAT (Large et al., 2015; Tal et al., 2017; Large et al., 2023), what renders syncopation especially interesting is that, despite being a deviation from metric expectations, it is a deviation that listeners chose not to endorse as evidence for revising their expectations (see also Velasco and Large, 2011): “Syncopation depends not only on listeners holding fast to the previously established metric context but also on their selectively construing what they hear, not as new invariants, but rather as subordinate to an established meter. Composers have long counted on (and exploited) our proclivity to maintain an established metric framework, and the force with which we will impose metric order on an uncooperative musical surface” (London, 2012, p. 16–17). London suggests that, to capture and maintain the attention of the listeners, music must challenge their metric expectations, at least to some extent, and that syncopation is one of the instruments to achieve such goal: “Music often depends on our making an effort to project and maintain an established meter in passages that involve things like syncopation and hemiola” (London, 2012, p. 25). In the framework of DAT, this effect is strictly related to the listeners’ sense of ownership and awareness of their own metric expectations: “local perturbations […] often make the listener more aware of their role in creating meter, as they must ‘feel a beat that is not there’ or otherwise have a palpable sense of the conflict between the music’s rhythms and their own metrical entrainment” (London, 2012, p. 88).

DAT, therefore, provides a framework for capturing the connection between syncopation and entrainment in the measure that syncopation affects the oscillatory nature of the attending mechanism that, according to DAT, are at the foundation of the notion of meter. The role of syncopation, according to this view, is that of awakening in the listeners a sense of awareness of their metric expectations and a sense of agency in establishing such expectations.

#### 3.1.3 Syncopation in PC

Similarly to DAT, PC regards syncopation as the occurrence of a musical event that contradicts the meter – conceived in PC, as we saw, as a set of predictions of different, hierarchically organized temporal resolutions – although not strongly enough to undermine the meter and force a revision of its predictions (Koelsch et al., 2019; Senn et al., 2019; Sioros et al., 2022; Stupacher et al., 2022). This view is supported by significant neurological evidence (Vuust and Witek, 2014, p. 6–7). Even more interesting for the goals of the present contribution is how the PC model of meter is able to model the inverted U-shape relationship between syncopation and affective and bodily entrainment as a function of its hierarchical organization and the bidirectional flow of information across its layers: “The inverted U-shape found between degree of syncopation in groove, wanting to move, and feelings of pleasure can be seen as complying with a hierarchical perceptual system at its higher and more subjectively manifested levels. At this higher level, prediction in perception and action facilitates affective and embodied experiences. At low degrees of syncopation, there is little incongruence between the rhythm of the groove (the input) and the meter (the predicted metrical model). Thus, little prediction error is being fed forward from the lower to the higher levels, and the experiential effect is weak – there is little pleasure, and little desire to move. At high degrees of syncopation, the degree of complexity is so high, and the rhythmic input deviates from the metric framework to such an extent, that the predicted model breaks down. Affective and embodied responses are decreased since the system is in the process of ‘learning’ and adjusting its internal models. Also here there is little prediction error since the brain is unable to provide an appropriate prediction model to compare the incoming input with” (Vuust and Witek, 2014, p. 9).

Indeed, this view calls for an explanation of the correlation between the predictive nature of meter on the one hand and the mechanisms of bodily entrainment and pleasure on the other (Meyer, 1956; Huron, 2006). As for what concerns pleasure, a possible explanation contemplated by Vuust and Witek (2014, p. 10) is based on Clark’s (2013) take on PC whereby the goal of the brain is not that of minimizing prediction error but, rather, that of maximizing prediction success. In the context of this assumption, it is entirely plausible that the brain may in fact reward prediction error, particularly at lower levels, because it leads to learning – that is, to the production of more successful predictive models at higher levels. Preliminary evidence from research in rodents suggests that this effect is plausibly mediated by dopamine in the mesolimbic pathway (Gebauer et al., 2012). In particular, Schultz (2007) and Schultz et al. (2008) report dopamine release in rodents to both expected and unexpected stimuli and suggest that dopamine may play a role in ensuring “a balance between ‘explaining away’ prediction error in the short term, and maintaining an incentive to engage in novel activities (of potential high risk) leading to adaptive learning in the long term” (Vuust and Witek, 2014, p. 10).

As for what concerns desire to move that is typically associated with syncopated rhythms, a plausible hypothesis^10^ is that it is a consequence of the fact that syncopated rhythms present prediction violations that cannot be resolved on the basis of auditory information alone. Studies have demonstrated that in conditions of auditory ambiguity, the motor system may help disambiguating the perceptual input. In PC models, it may well be plausible to interpret the motor involvement as internal simulation (Skipper et al., 2017; Proksch et al., 2020). Bodily entrainment would operate, therefore, as a mean to disambiguate an otherwise ambiguous auditory stimulus (we will discuss this hypothesis further in Section 5).

## 4 Syncopation as structure bootstrapping

In this section, we present an alternative approach to syncopation whereby syncopation is a strategy of structure bootstrapping – that is, a strategy that exploits the linear order of musical events to express the presence of an underlying hierarchical organization. This view is not meant to be incompatible with the theories reviewed in the previous section. Rather, in our intentions, it discloses an aspect of syncopation that may further explain its musical function and value.

### 4.1 Bootstrapping temporal predictions from IOI’s

We take inspiration from the Bootstrapping Principle (BP) discussed in Section 2.3.4 to propose a cognitive model of syncopation whereby syncopation represents a strategy to express the containment of lower level temporal predictions within higher level temporal predictions on the sole basis of IOI’s.

**Figure 7:**
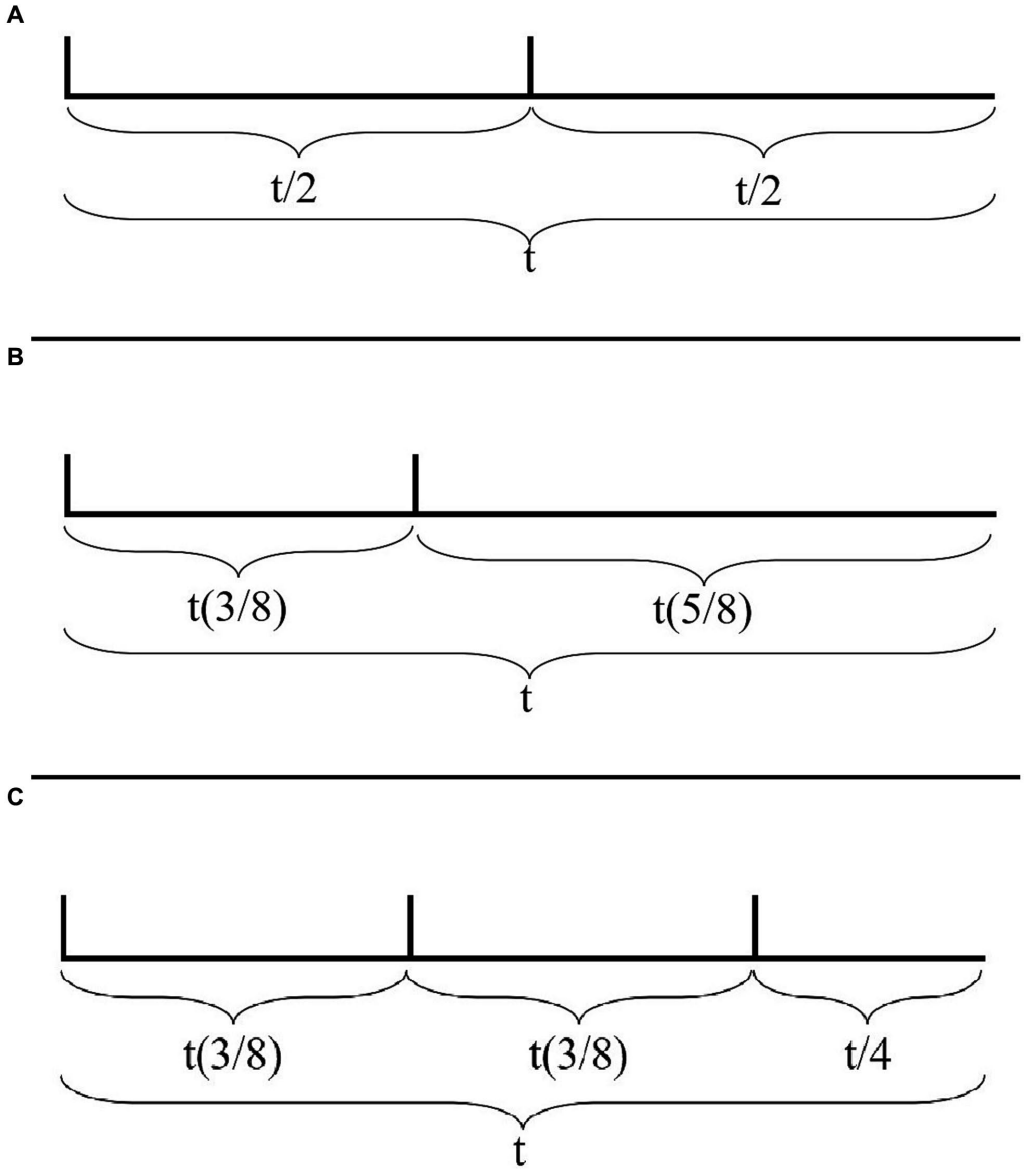
**(A)** A rhythm represented solely as (onsets of) attacks on a cyclic timeline of duration *t*, with its IOI’s relative to *t*. The rhythm consists of the cyclic repetition of two attacks evenly distributed within *t*, therefore with IOI’s of duration *t*/2. **(B)** A rhythm represented solely as (onsets of) attacks on a cyclic timeline of duration *t*, with its IOI’s relative to *t*. The rhythm consists of two attacks with IOI’s of *t*(3/8) and *t*(5/8). **(C)** The tresillo rhythm represented solely as (onsets of) attacks on a cyclic timeline of duration *t*, with its IOI’s relative to *t*. The rhythm consists of three attacks with IOI’s of *t*(3/8), *t*(3/8), and *t*/4.

Consider the two rhythms in Figures 7A,B. These rhythms are represented solely as (onsets of) attacks on a cyclic timeline of duration *t*, with their IOI’s relative to *t*. The rhythm in Figure 7A consists of the cyclic repetition of two attacks evenly distributed within *t*, henceforth with IOI’s of duration *t*/2. The rhythm in Figure 7B (which corresponds to the Charleston rhythm) differs from that in Figure 7A in that the second attack has been anticipated by *t*/8.

Consider, then, what the simplest predictive frameworks are that can be bootstrapped by the listener for the two rhythms on the sole basis of the IOI’s between the attacks. Indeed, other factors, such as timbre, pitch, or other contextual factors, may play a role in suggesting that the repetition has to be interpreted as a cycle of two attacks one of which is more prominent than the other in the intended metric organization. Furthermore, it has been demonstrated that there is a natural cognitive tendency to group isochronous consequent attacks in binary structures (Brochard et al., 2003). Once again, our goal is to evaluate how IOI’s alone contribute metric information, irrespectively of other factors.

In the case of the rhythm of Figure 7A, the listener is confronted with the regular repetition of an attack every *t*/2. IOI’s alone provide no evidence for any hierarchical relation between the two attacks in the sequence. Notice furthermore that the surface rhythm suggests an underlying pulsation that has a pulse every *t*/2 and does not suggest a pulsation that is more fine-grained than that (also in this case, a more fine-grained pulsation may be suggested by other factors but, crucially, not IOI’s alone).

Conversely, the predictive framework required by the rhythm in Figure 7B is significantly richer. To begin with, the ratio between the two attacks occurring within the cycle *t* demands an underlying isochronous pulsation that divides *t* in (at least) eight pulses.^11^ Furthermore, the asymmetric relation between the two attacks makes it so that any potential prediction that can be made at the lowest temporal resolution (from one attack to the next) must be discarded in favor of a prediction of a larger temporal resolution (each attack will occur every eight pulses). In this case, therefore, IOI’s alone provide sufficient information to project a metric structure that recurs every eight pulses.

Consider, then, an equivalent representation of the tresillo rhythm (Figure 7C). As the figure demonstrates, the IOI ratios of the tresillo demand an underlying isochronous pulsation of (at least) eight pulses. Notice, furthermore, that the first two IOI’s suggest a low level prediction (from one attack to the next) of *t*(3/8). That is, the IOI’s detected between the first three attacks provide evidence for predicting that a fourth attack will occur again after *t*(3/8). This low level prediction, however, is falsified by the IOI between the third and the first attacks. A coherent predictive framework can be maintained, on the face of this failure, by bootstrapping a higher level prediction that the entire cycle will repeat after an interval *t*.

To see this point more concretely, consider how a rhythm such as the tresillo could be implemented mechanically by using a stack of two step-sequencers, that is a mechanism (as is found in the sequencing component of many synthesizers) that sends trigger signals at regular time intervals. Suppose a step-sequencer that runs at steps of duration *t*/8 is made to cycle through three steps and to send a trigger signal on the first step that triggers an attack. Suppose then that a second step-sequencer, also running at steps of duration *t*/8, is made to cycle through eight steps and to send a trigger signal on the first step that triggers an immediate reset of the first step-sequencer. The result will be the tresillo rhythm.

As the example demonstrates, the tresillo can be conceived as a low-level prediction that an attack will occur every three pulses that is contained within a higher-level prediction of eight pulses, where containment means that the lowerlevel prediction is overruled by the higherlevel prediction.

This strategy can be pushed even further with a rhythm such as the cinquillo, which can be conceived as a low-level prediction that an attack will recur every two pulses that is contained within a higher-level prediction of three pulses that is in turn contained within an even higher prediction of eight pulses.

Importantly, both in the case of the tresillo and the cinquillo, the asymmetric distribution of their attacks within the repeating cycle provides a sufficient condition for bootstrapping not only predictive layers that go beyond the low-level predictions from one attack to the next but also relations of prominence between the attacks. In the case of the tresillo, for example, the relation of containment between the levels allows the listener to identify the first attack as the most prominent – the one, that is, where the onsets of both predictive layers coincide.

Both examples demonstrate how, by its ability to create asymmetric distributions of attacks within a rhythmic cycle, syncopation is in fact a powerful tool for informing the bootstrapping of a higher-level predictive structure on the basis of the sole IOI’s between the successive attacks occurring in the cycle.

More technically, in the context of rhythm perception, we can think of the Bootstrapping Principle (BP) as a strategy that attempts at resolving prediction errors by adopting larger chunks of IOI’s as the relevant predictor. The parser first attempts at making predictions considering single IOI’s as the relevant prior. Whenever the evidence provided by the stimulus is inconsistent with the prediction, the parser tries to make predictions by considering sequences of two IOI’s as the relevant prior. The process proceeds to larger and larger sequences of IOI’s until the prediction error is eliminated.^12^

To see this methodology at work, consider the simple un-syncopated rhythm in (8) and compare it with its syncopated version (9).

(8)a.1010

b.[2-2]

(9)a.1100

b.[1-3]

In the case of (8), the first evidence provided by the stimulus to the parser is an IOI of time *t* = 2. The prediction made is, therefore, that the following IOI will also be of time *t* = 2. This prediction is met and the parser has no reason to apply BP.

In the case of (9), the first evidence provided by the stimulus to the parser is an IOI of time *t* = 1. The prediction made is, therefore, that the following IOI will also be of time *t* = 1. This prediction is not met as the following IOI is of time *t* = 3. Given BP, the parser replaces its prior with the sequence of the preceding IOI’s, hence a sequence of one IOI of time *t* = 1 and a second IOI of time *t* = 3.

As it can be seen, the asymmetric distribution of attacks in the syncopated rhythm (9) triggers the implementation of BP and, with it, the formulation of predictions of a larger temporal resolution than that of the individual attacks and their IOI’s.

It should be observed that the strategy devised in this section has a broader application than that of syncopation as we have defined it so far. As we saw, syncopation is generally defined as the occurrence of a musical event on a metrically weak position preceding a rest on a metrically strong position (Huron, 2006). However, the strategy we have presented in this section extend also to cases of so-called fourth-position syncopation or anticipation (Temperley, 2021) such as (10).

(10)Fourth-position syncopation (anticipation)

a.1001

b.[3-1]

### 4.2 Accounting for the musical properties of syncopation: meter as a model of bodily entrainment

Based on the observations above, in this section we advance the proposal that syncopation is such a powerful rhythmic device across musical cultures (Toussaint, 2013; Witek et al., 2020) and so successful in eliciting the urge to move precisely because of its ability to inform the bootstrapping of hierarchically richer metric structures.

This proposal involves a revision of the more traditional understanding of the notions of syncopation and, more generally, meter. As we saw, syncopation is canonically defined as the occurrence of a musical event on a metrically weak pulse preceding (or following, if we also consider fourth-position syncopation) a rest on a metrically strong pulse. This definition offers an understanding of syncopation that is top-down and negative. Top-down in the sense that it defines syncopation in terms of the distribution of a musical event within an established metric framework, whose role is constraining (or predicting) the occurrence of musical events. Negative in as much as it defines syncopation in terms of a violation of the constraints (or predictions) of an established metric framework. Once syncopation is defined in such a top-down negative manner, it becomes inevitably difficult to account for its productive role in eliciting bodily entrainment and popularity across musical idioms. In fact, it becomes difficult even to explain why syncopation should exist at all.

In contrast, we propose a view of syncopation that emphasizes its bottom-up and constructive dimensions: Bottom-up in the sense that it understands syncopation in terms of how the linear distribution of IOI’s in a rhythm supports the inference of metric information, rather than the other way around; constructive because it regards syncopation as a source of metric information rather than a violation of it.

A major implication of this perspective is that it is incompatible with the view that meter is an model of rhythm – that is, a set of constraints or predictions governing the occurrence in time of musical events. Rather, if we are correct, meter is a model of temporal regularities that is *informed by* musical rhythm. That is, it is not meter that governs the linear shape of rhythm but the linear shape of rhythm that informs meter.

Indeed, this raises the question of what meter is a model of if it is not a model of musical rhythm. We propose that meter is, in fact, a model of bodily entrainment – a model of recurring regularities that listeners extrapolate from the musical surface in order to entrain motorically with it. Syncopation is, henceforth, a trigger for the extrapolation of such regularities because of its capacity to elicit the application of BP.

The significance of the connection between rhythm and movement in music perception has been studied since Fraisse (1963, 1978, 1981, 1984; see Clarke (1999) for an overview) and has represented an important source of evidence for embodied theories of musical cognition – such as Borgo (2005), Clarke (2005), Cox (2016), De Souza (2017), and Leman (2007). In recent years, a number of neuro-functional studies have demonstrated the association between meter perception and the activation of motor areas even in the absence of any overt movement (Schubotz et al., 2000; Chen et al., 2006; Grahn and Brett, 2007; Zatorre et al., 2007; Chen et al., 2008; Bengtsson et al., 2009; Grahn, 2009; Grahn and Rowe, 2009, 2012). In a study of the neural response to complex rhythms, Chapin et al. (2010) found that selective attention to syncopated rhythms led to increased BOLD responses in “motor areas including left SMA, right basal ganglia (caudate, globus pallidus, extending into nucleus accumbens), and left postcentral gyrus (BA 3)” (p. 7) – although such activity was observed only after three repetitions of the rhythms. These results find an immediate explanation within the framework we propose: The activation of motor areas is expected given the hypothesis that meter is a model of bodily entrainment, rather than a model of rhythm perception; the fact that three repetitions of a syncopated rhythm are necessary for the activation of motor areas is expected given the proposed bootstrapping mechanism, which requires the detection of cyclicities of a higher structural level, and the fact that a model of bodily entrainment is available only once these higher-level cyclicities have been detected and sufficiently confirmed.

Fitch (2016) reviews a number of biological reasons, both neurological and ecological, that movement and dance are central factors of musical rhythm and argues that “important rhythmic aspects of […] music, especially meter and syncopation, cannot be properly understood without reference to movement and dance, and that the persistent tendency of ‘art music’ to divorce itself from motion and dance is a regrettable phenomenon to be resisted by both audiences and theorists” (p. 1–2). In our framework, the connection between rhythm and movement follows from the hypothesis that meter is a model of bodily entrainment informed by the musical surface. The special role played by syncopation in this relationship has to do with its ability to inform structurally richer meters – that is, structurally richer models of bodily entrainment. In a sense, if the role of rhythm is to induce cyclic movement, the role of syncopation is to induce more complex cyclic movement – movement, that is, that has a richer inner organization.

Especially informative, in this respect, is a study by Naveda and Leman (2009) of samba music and dance. The study is grounded on the hypothesis that music and dance ought to be regarded a holistic phenomenon in samba culture (Sodré, 1979; Browning, 1995) and the observation that the distinctly syncopated rhythms of samba music, which characteristically include rhythmic forms such as the tresillo and the cinquillo (Sandroni, 2001), are pivotal to eliciting (a desire for) movement in the listeners – what Browning (1995, p.9) calls the ‘hunger’ for movement. To test the hypothesis, Nevada and Leman develop a method to investigate periodicities in both samba music and dance and apply it to a set of samba dance performances and the music over which they were performed. Their cross-modal approach is based on the Periodicity Transform (PT) method developed by Sethares and Staley (1999, 2001), which is an analytic method that searches for periodic events in a signal on the basis of a predefined heuristic. An innovative aspect of Nevada and Leman’s approach is that they use, as heuristic for the PT analysis of both music and dance, the musical meter of samba music. This is a binary meter, with accentuation on the second beat (Salazar, 1991; Chasteen, 1996; Mariani, 1998; Sandroni, 2001). The surprising result of their analysis is that “the binary metre of samba music, which is strongly recognized within musicology, is much more evident in the results of metrical content of dance than in the apparent ambiguous metre of the auditory stream” (p. 271). In fact, the analysis demonstrates how the highly syncopated rhythmic forms of the music fail to a significant extent to meet the periodicities of the assumed meter whereas, in contrast, the same metric periodicities are evident in the dancers’ movements. The authors take these results to suggest that “perception of samba may be movement-based in the sense that through self-movement (of the dancer in response to music) musical patterns get rhythmically disambiguated” (p. 272) and that this process of disambiguation “may well be the effect of the biomechanics of the human body, which imposes a certain motor structure onto an ambiguous auditory stimulus” (279). This line of reasoning can be taken even further under the view proposed here that meter is not a model of rhythm but a model of bodily entrainment. If we abandon the view that the role of meter is that of governing/predicting the occurrence of musical events and, instead, maintain that it is the role of musical events to induce meter as a model of bodily entrainment, then it is less surprising to find that meter in a highly syncopated musical form such as samba is more evident in its embodiment than it is in its musical surface.

The view of syncopation proposed here makes it legitimate to ground meter on principles belonging to the biomechanics of the human body. Consider again the example of the tresillo. As pointed out, the tresillo is typically understood in the context of a binary meter. To explain this in our model, it is sufficient to supplement the bootstrapping strategy suggested in the previous section with a tendency, belonging to the realm of motor entrainment rather than that of musical rhythm, toward entraining motorically to isochronous cyclicities. From the perspective of the bottom-up bootstrapping procedure adopted, realizing a cyclicity that is divided isochronously requires first identifying a predictive cycle that is not contradicted by a higher-level prediction – what London (2012) calls the N-cycle. In the case of the tresillo the lowest predictive level that is not contradicted by a higher-level prediction is the cycle of duration *t* in Figure 7C. As we saw, the syncopated distribution of the attacks in this cycle demands a division of the cycle of length *t* into eight pulses. Hence, if a principle of isochronous bodily entrainment is to apply successfully, it must divide the cycle binarily (a pulsation of eight pulses can be divided by two but not by three). According to this view, the meter associated to the tresillo is a model of how to entrain with it that is further optimized for isochronous bodily entrainment.^13^

Ultimately, according to our proposal, syncopated rhythms do not violate metric expectations. The mismatch between rhythm and meter is rather a consequence of the fact that the processes that determine the model of bodily entrainment within the N-cycle of a rhythm, which have to do with the biomechanics of human movement, may be distinct from the process that supports the bootstrapping of the relevant N-cycle from the musical surface of the rhythm.

## 5 Discussion

As anticipated, our proposal is not to be regarded as incompatible with existing accounts of syncopation. On the contrary, we believe that it provides further support to frameworks such as PC and DAT and, in fact, may further substantiate some of their insights. In particular, if our hypothesis is correct, it supports the view that syncopation, while causing the breakdown of low-level predictions, is nonetheless appreciated by the listeners because of its capacity to provide evidence for the bootstrapping of predictions of higher temporal resolution. It is indeed a type of low-level prediction error that promotes the learning of higher-level generalizations and, with it, the induction of richer predictive models.^14^

Our framework differs from the other models of meter and syncopation, particularly GTTM, in that it places its emphasis on the bottom-up relation between rhythm and meter and rejects the view of meter as governing (or making predictions about) rhythm. As we saw, according to our proposal, meter does not constraint rhythm but guides bodily entrainment. Importantly, this view does not exclude the occurrence of top-down processes that limit the range of the possible rhythms that can occur in the context of an established meter. However, it does substantially limit the scope of what is to be regarded as a rhythm that violates an established meter. More precisely, according to our proposal, a rhythm can be said to violate an established meter only when it contradicts the bootstrapping process that has produced the established meter and, as a consequence, requires a revision of the model of motor entrainment adopted.^15^ This flexibility may be of help in capturing Temperley’s (1999, p. 34–35) observation that, in the context of rock music, syncopation is often exploited by performers and composers as a tool to provide rhythmic variety within a stable metric context. It is also important to observe that, although our proposal rejects the view of meter as making predictions about the occurrence of musical event, it does not mean that meter is not a predictive model in other respects. It is, as we saw, a model of bodily entrainment. Hence, it makes predictions about how to entrain motorically with a rhythm. This view is confirmed by phenomena such as the negative mean asynchrony (NMA), a phenomena observed by a number of studies whereby listeners who are asked to tap their finger to a rhythm tend to anticipate musical events with their movements (see Repp and Su, 2013, for an overview).

Our framework also differs from the other accounts in the way it explains why syncopated rhythms are so popular and effective in eliciting bodily entrainment. It differs from GTTM in that it does not regard syncopation as a source of tension but, rather, as a source of metric information and it differs from DAT in that it does not rely on the sense of agency of interpreter. As we saw, a plausible hypothesis in the context of PC is that bodily entrainment is a strategy to disambiguate otherwise ambiguous syncopated rhythms. In our view, this hypothesis encounters three limitations.

The first is that the activation of motor areas while attending to rhythmic sequences is not an exclusive effect of syncopated rhythms. Listening to all rhythms, including non-syncopated ones, activates motor areas. For example, Grahn and Rowe (2012) find that even “perception of a regular pulse, or beat, within a sequence of temporal intervals is associated with basal ganglia activity.” What is characteristic of syncopated rhythms is that they have a significantly more pronounced effect on motor areas. The hypothesis that bodily entrainment is a disambiguation strategy for ambiguous rhythms would not explain why bodily entrainment is detected, at least neurologically, with non-ambiguous rhythms.

A second issue concerns the status of syncopated rhythms as ambiguous rhythms. In particular, it is not clear that the distinction between metrically ambiguous and metrically unambiguous rhythms overlaps with that between syncopated and non-syncopated rhythms. On the one hand, there are non-syncopated rhythms that are decisively ambiguous. A simple isochronous sequence of identical attacks is compatible with a number of metric analyses. On the other hand, there are syncopated rhythms that are not ambiguous. As we saw, when we focus on the exact process of how predictions are bootstrapped from the surface of the tresillo rhythm, we find that the asymmetric distribution of attacks in the rhythm lends itself to an optimal analysis whereby a prediction that an attack will occur every three pulses is overruled by a prediction, of larger temporal resolution, that the embedded sequence will repeat every eight pulses. While it is possible to conceive of alternative analyses (based for example on different rotations of the rhythm), they are not as optimal and simple as this one. From this perspective, an isochronous sequence of identical attacks is, in fact, more ambiguous than a syncopated one in the sense that it imposes less of a constraint on the set of possible metric analyses. Conversely, syncopated rhythms are more restrictive in delimiting their analytic space precisely in virtue of the asymmetric distribution of their attacks. Also relevant to this issue, are the arguments presented by Temperley (1999) against the view suggested by Lerdahl and Jackendoff’s (1983) that syncopation is a means for introducing metric ambiguity and, with it, tension. As discussed in Section 3.1.1, Temperley presents evidence that syncopated phrases in rock music are not perceived as ambiguous or as inducing a sense of tension (see also Keller and Schubert, 2011).

The third issue concerns the study of Chapin et al. (2010) who found an increased BOLD responses in the basal ganglia only after three repetitions of a syncopated rhythm. If bodily entrainment was a disambiguating strategy, we would expect that motor areas would engage when the ambiguity was detected, that is, as soon as an event would contradict the current model, and disengage when the ambiguity was resolved, that, is as soon as the model would be updated as to encompass the unaccounted event. Conversely, our framework predicts that motor areas engage as soon as a meter has been bootstrapped from the rhythm.

The main difference between our framework and PC is thus that for PC bodily entrainment is an instrument to compensate a metric ambiguity whereas, according to our proposal, it is syncopation that is an instrument to enhance bodily entrainment by lending itself to providing a richer cyclic temporal organization. As said, we find our view to be otherwise entirely compatible, and, in fact, reliant upon PC’s notion of a bidirectional predictive system that has a hierarchical structure and its analysis of syncopation as a low-level prediction error that informs higher levels. In this respect, our model specifies some inherent features of the PC model that have so far remained underspecified – more particularly, the mechanisms that mediate the exchange of predictive potential between the hierarchical levels and the role that syncopation plays in them.

It is finally important to stress once more that the framework we have proposed offers a decisively limited account of syncopation as a broader musical phenomenon. As already mentioned, our approach focuses exclusively on rhythm in terms of IOI’s and disregards at least three classes of factors that are central to a broader understanding of syncopation. The first concerns factors of musical context. In the reality of musical practice, rhythms are not only IOI’s but are produced by instruments with all sorts of timbral, melodic, and harmonic properties. These factors play a decisive role in syncopation, as attested, for example, by Temperley’s (1999) discussion of how syncopation in the lyrics of rock music is significantly affected by the phonological and prosodic properties of the words that are sang. Furthermore, rhythms are rarely performed in isolation. In poly-instrumental performances, different instruments play different, interrelated rhythms and provide together a rhythmic texture rather than a single rhythmic figure. In cases such as these, one instrument may use the support of the metric context provided by other instruments to play highly syncopated rhythmic figures.

The second class of factors has to do with cultural context. As stressed in the literature (Jones, 1990; London, 1990), factors related to the specificity of a musical idiom play a crucial role in guiding the listener’s perception of the meter underlying a rhythmic surface. Huron and Ommen (2006), for example, detect an increase in the amount of syncopation in American popular music between 1890 and 1939. Similarly, Kirlin (2020) demonstrates that syncopated patterns in rag-time music increment significantly from the early days of the musical style (1890’s) to its later days (1920’s). Arguably, as the metric conventions of these styles became more culturally ingrained, composers were able to enjoy more rhythmic freedom.

The third factor concerns the phenomenon of subjective rhythmization (Bolton, 1894; Fraisse, 1982), “whereby sounds of a monotone metronome sequence are experienced as having different intensity and that these intensity differences follow a regular pattern. In other words, despite the sounds having objectively equal amplitude, they are perceived as subjectively different” (Bååth, 2015, p. 244). One possibility to integrate these factors into the proposed framework is to admit that the bottom-up strategy based on BP co-exist with top-down constraints of either contextual, cultural, or cognitive origin. A more appealing possibility from the perspective we have been advocating for consists in reformulating these factors as themselves bottom-up strategies of structure projection that may co-exist and possibly compete with BP.^16^ More technically, this would amount to expressing these factors as strategies affecting the predictive priors of the parsing mechanism.

While we leave a full-fledged development of this hypothesis to future research, we briefly speculate on how this perspective may be applied to the phenomenon of subjective rhythmization. The phenomenon can be accounted for by a propensity on the part of listeners, whose source may be intrinsically cognitive or cultural, to project hierarchical structure at all costs, that is, even in absence of evidence in the surface rhythmic structure for doing so. In turn, this propensity, may be formalized in terms of a bootstrapping mechanism that allows assuming larger sequences of IOI’s as a predictive prior provided doing so does not produce prediction errors.

### 5.1 Some considerations on the relationship between music and language

We conclude by discussing our proposal in the broader context of the relationship between language and music.

The relation between language and music has become the subject of growing interest in recent years, encouraged by the emergence of novel evidence that the neurology and cognition of the two domains feature notable overlaps (Patel et al., 1998; Steinhauer et al., 1999; Koelsch et al., 2002; Levitin and Menon, 2003; Tillmann et al., 2003; Koelsch et al., 2004; Knösche et al., 2005; Brown et al., 2006; Patel, 2008; Rauschecker and Scott, 2009; Kotilahti et al., 2010; Schön et al., 2010; Abrams et al., 2011; Rogalsky et al., 2011). There are different views on the neurological and behavioral evidence of a cognitive overlap between language and music. These range from skeptical ones, advising against overstating the overlap, to more optimistic ones. At one end of the spectrum, Jackendoff (2009) has insisted that while “[l]anguage and music share a considerable number of general characteristics and one detailed formal one, namely metrical structure […] most of what they share does not indicate a particularly close relation that makes them distinct from other cognitive domains” (p. 203). At the other end, Patel’s (2012) shared syntactic integration resource hypothesis (SSIRH) contends that music and language originate from a shared set of neurobiological resources and begin their life in human cognition by relying on a common neural network although, as the developing brain is exposed to novel inputs, it ‘prunes’ out inefficient connections and ends up establishing two separate pathways that share a pool of cognitive features. An even stronger stance is that of Katz and Pesetsky’s (2011) Identity Thesis for Language and Music (ITLM), according to which “all formal differences between language and music are a consequence of differences in their fundamental building blocks (arbitrary pairings of sound and meaning in the case of language; pitch-classes and pitch-class combinations in the case of music). In all other respects, language and music are identical” (p. 3). This hypothesis, motivated by theoretical considerations concerning the structural properties of both music and language, maintains that language and music share a common syntactic component and that their differences are due to the fact that the same set of combinatorial rules is applied to different lexicons of primitive building blocks.

#### 5.1.1 Rhythm and language

Jackendoff (2009) identifies rhythm (particularly metrical structure) as the domain where music and language are most closely related. Indeed, rhythm is central to both music and speech. Both speech and music are perceived as sequences of sound events that unfold in time and are organized around a binary distinction between strong/stressed and weak/unstressed tones, although it is important to keep in mind that musical meter and meter in language are fundamentally distinct as only musical meter is based on a regular pulsation.

There are only a few studies testing the association between the perception of rhythm in music and speech. A first class of studies focuses on the rhythmic perceptual skills of healthy adults. Marie et al. (2011) report that musicians perceive the metric structure of words more accurately than non-musicians and that incongruous syllable lengthening elicit stronger ERP activations in musicians. Cason and Schön (2012) report that priming with rhythmic tones can enhance the phonological processing of speech. Gordon et al. (2011) demonstrate that synchronizing musical meter and linguistic stress in songs can enhance the processing of both lyrics and musical meter. A second class of studies concerns the rhythmic perceptual skills of populations with language impairments. Difficulties in auditory rhythmic processing have been reported in children with reading disabilities and other language deficits (Corriveau et al., 2007; Corriveau and Goswami, 2009; Goswami et al., 2010; Goswami, 2012). Huss et al. (2011) report that sensitivity to musical meter is a successful predictor of phonological awareness, which, in turn, is a crucial precondition to reading development. Significant in this respect is also the observation by Alcock et al. (2000) that the members of the KE family, who suffer from a genetic developmental disorder in speech and language, experience problems also in the perception and production of rhythm. Finally, a number of experiments conducted by Wolff (2002) demonstrate that dyslexics adolescents are significantly less accurate than their peers with typical reading skills in tapping along the regular beat of a metronome.

As discussed in the previous section, the proposal put forward in this contribution to account for rhythmic syncopation is inspired by a parallel with natural language syntax, in particular, with the Bootstrapping Principle (BP) proposed by Delfitto (2023) and Vender et al. (2023) as a mapping from the linear order of words to hierarchical structure. The hypothesis, in this respect, suggests a view of the relation between music and language in the spirit of Katz and Pesetsky’s ITLM. In particular, it suggests that music and language share a common syntactic component that supports the bootstrapping of hierarchical relations from relations of linear order (precedence in the case of language and distance in terms of IOI’s in the case of rhythm) but applies this common syntactic strategy to different primitive elements.

In this respect, it is worth pointing out that asymmetric structures and configurations are pervasive in language (Di Sciullo, 2003a,b, 2017b), to the point of being regarded as “part of the initial state of the language faculty, enabling human beings to develop the grammar of the language to which they are exposed, to interpret and to quickly generate the expressions of this language in a relatively short period of time” (Di Sciullo, 2003a, p. 3). We have already discussed the notion of asymmetric c-command and its role in theories of linearization such as Kayne’s LCA (Kayne, 1994). Asymmetric relations based on c-commands have also been adopted to account for syntactic phenomena such as movement (Ross, 1967; Chomsky, 1981; Rizzi, 1990), agreement (Chomsky, 1998; Di Sciullo, 1999) and phenomena at the syntax-semantics interface, such as anaphora, operator-binding, and the scope of logical operators (Reinhart, 1976, 1983; Chomsky, 1981; Kayne, 1984; Higginbotham, 1985; Aoun and Li, 1993; Hornstein, 1999; Di Sciullo, 2012). Asymmetry is also pervasive in phonology and morphology (Van Der Hulst, 1984; Baker, 1988, 1998; Rice, 1992; Dresher and Rice, 1993; Rice and Avery, 1993; Keyser and Roeper, 1997; Dresher and van der Hulst, 1998; Piggott, 1999, 2000; Raimy, 2000; Van Der Hulst, 2000; Di Sciullo, 2005). Di Sciullo (2005, 2017a) has developed models of morphological, syntactic, and semantic language learning and competence grounded in asymmetry-based parsers. Fundamental asymmetries have also been detected in language typology (Hawkins, 2002). Dehaene (2010) demonstrates the role of asymmetry in reading.

An especially illustrative case-study is offered by Domahs et al. (2008) and Henrich et al. (2014) in the domain of metrical phonology.^17^ Some languages (such as English and German) have the possibility of reversing the stress pattern of a world in order to avoid the occurrence of two immediately adjacent stressed syllables. For example, the English word *thirteen*, whose lexical stress is on the second syllable, becomes *thìrteen* in *thìrteen mén*. Most approaches to this phenomenon (Liberman and Prince, 1977; Hayes, 1984; Selkirk, 1986; Speyer, 2010) agree that stress shifts respond to a Principle of Rhythmic Alternation (PRA) that requires a consistent alternation between stressed and unstressed syllables. Such rhythmic alternations have been demonstrated to play a role in aiding speech production (Tilsen, 2011) and perception (Cutler and Foss, 1977), early language acquisition (Jusczyk, 1999; Nazzi and Ramus, 2003), and memorization (Bolinger, 1958; Auer and Uhmann, 1988). As observed by Henrich et al. (2014), it is notable that stress-shifted word precedes the shift-triggering word. This means that the legitimacy of the stress-shifted word cannot be validated when it is encountered (as further confirmed by the ERP results of Henrich et al., 2014) but only later, when the shift-triggering word is encountered. In our terms, we can say that the stress-shifted word projects a prediction that the following word will begin with a stressed syllable, hence projecting a metrical-phonological constituent structure that spans beyond the word level.

If our perspective is correct, the pervasive presence of configurational asymmetries in language follows from the bootstrapping mechanism BP, which, as we saw, maps linear order into hierarchical structure. Asymmetries in phonological, morphological, syntactic, and semantic categories are a necessary ingredient for detecting in the linear order elements that are projected by BP into larger constituent structures. Asymmetric configurations in language are, therefore, the parallel of syncopation in musical rhythm, in that, by their very nature, guide and constraint the projection of hierarchical structure.

Recent neurological evidence brings further support to the proposed perspective. Heard and Lee (2020) perform a meta-analysis to identify brain regions that are consistently implicated across studies of both musical rhythm and natural language syntax. The studies included in this meta-analysis examine aspects of musical rhythm cognition such as rhythm perception, beat detection, and meter and aspects of syntax such as merge (the simplest combinatorial process of syntax; Chomsky, 1995), movement (the displacement of heads and constituents from the position in the syntactic structure where they are originally merged, e.g., fronting of *wh-* elements in question), and garden-path effects that require reanalysis (e.g., “the horse past the barn felt”). The results of the meta-analysis demonstrate that “rhythm mostly recruited symmetrical clusters in bilateral cortical and subcortical areas including the IFG, putamen, SMA, STG, insula, and IPL. Syntax predominantly engaged a left-lateralized network including the IFG, PMC, STG, insula, and IPL. Overlap between rhythm and syntax was found in the left IFG, left SMA, and bilateral insula. Additional intersections between rhythm and each syntax process yielded clusters within a similar part of the left IFG (pars Opercularis), but only movement and reanalysis recruited motor regions such as the SMA” (Heard and Lee, 2020, p. 6).

Particularly notable from our perspective is the observation that motor regions were activated by those syntactic tasks that demand particular effort in identifying constituent structure on the basis of linear order. In the case, of movement, identifying constituent structure requires reconstructing the position where the displaced elements was originally merged. For example, in the sentence “who did Mary meet?” the parser must reconstruct “who” as the direct object of “meet.” Garden-path sentences are sentences that require re-analysis by considering a lager chunk of structure. For example, in a garden-path sentence such as “the horse past the barn felt,” the parser is first led to regard “barn” as the last word in the sentence. However, when it meets “felt,” it is induced to reject this analysis and consider a larger chunk of structure as the relevant constituent that qualifies as a sentence. As with syncopation, we observe a lower-level prediction error that pushes the parser to consider a larger structure as the relevant domain of analysis.

The authors further observe that “an important characteristic of music and language is the hierarchical organization of serial temporal information (Lashley, 1951; Fiebach and Schubotz, 2006; Fadiga et al., 2009; Jackendoff, 2009; Fitch and Martins, 2014; Jeon, 2014)” (Heard and Lee, 2020, p. 8) and “such action sequences are mainly mediated by frontal motor circuitries including the left IFG, PMC, and SMA (Koechlin and Jubault, 2006; Clerget et al., 2009). These frontal motor processors exhibited overlaps between rhythm and syntax in the present study, suggesting that they are also at play in analyzing the temporally unfolding hierarchies of musical rhythm and linguistic syntax. Relatedly, early left anterior negativities (ELAN) and parietal P600 components were observed when canonical structures were violated in action and language (Maffongelli et al., 2015)” (Heard and Lee, 2020, p. 9).

Although the deeper reasons underlying the connection between language and music perception, on the one hand, and the activation of motor-areas remain subject to scrutiny, Assaneo and Poeppel (2018) provide empirical and computational evidence that motor areas act (not just functionally but biophysically) as an interareal neural oscillator that directly entrains to regularities occurring in the stimuli within domain-specific time ranges.

In closing this section, it is important to point out that there remain significant differences between language and rhythm that are relevant to the theoretical perspective proposed. Firstly, it is important to point out that, in syntax, BP is not only meant to address the issues regarding the bootstrapping of hierarchical relations from purely sequential relations but also the issues around the categorial labeling of the progressively larger units created in the hierarchical domain. The rich lexicon of natural languages is organized around grammatical categories. The issue of labeling concerns how a constituent that results from the combination of two lower-level chunks belonging to different grammatical categories is assigned its own grammatical category and BP provides an algorithm for labeling as a function of linear ordering. Conversely, the lexicon of musical rhythm – in as much as it is conceived exclusively in terms of IOI’s – boils down to attacks that occur over time. In this respect, the natural question to be addressed, once we have established the existence of higher-level prediction layers, is whether labeling plays any role in hierarchical metrical space. We will briefly discuss this fascinating issue in the following section.

Another relevant difference between language and music is that meter is periodic whereas syntactic structure is not. From the bottom-up perspective contemplated here, the period of a meter is the smallest predictive cycle that is not contradicted by a higher-level prediction. This period acts as a cap on the application of BP. Once the smallest predictive cycle is identified, a model of bodily entrainment can be constructed and there is no need for further applications of BP. Conversely, the lack of periodicity in linguistic structure means that there is no necessary upper limit to the application of BP. The fact that syntactic complexity is potentially unbound in language reflects Humbolt’s insight that language “makes infinite use of finite means” and is at the foundation of generative approaches to syntax (Chomsky, 1965, p. 8). In top-down generative grammars, this property is captured by means of recursive rules that allow, for example, embedding of a grammatical category C within a larger constituent of the same category C (Hauser et al., 2002). We can capture the same facts by means of BP by not imposing any upper limit on its iterations when BP operates in language. Hence, from the current perspective, what is common to music and language is the core mechanism of BP. What is different is that, whereas in the context of metric rhythm the potential of BP is constrained by the need to identify a recurring metric pattern, in language it is not.

#### 5.1.2 Rhythm, hierarchy, and labeling

We have defined metrical space (in opposition to GTTM) not as an *a priori* hierarchical dimension to which rhythm must adapt but as a system of hierarchical solutions to which rhythm gives rise while optimizing predictive power (more in agreement with the bottom-up approaches of DAT and PC). Metrical structure is dynamically built, starting from a potentially infinite sequence of pulses, as a result of the effort of optimally capturing the recurrence rate of attacks in the sequence of pulses, that is, as a consequence of the effort of producing the optimal prediction concerning the distance in number of pulses between the onset of successive attacks (IOI). In our view, metrical structure is dynamically built by predicting IOIs over progressively larger intervals. This is essentially done by trying to identify the time interval that ensures the optimal prediction without being contradicted by a higher-level predictive cycle. Here, syncopation plays a crucial role. While its classical definition suggests it is an imperfection, it is in fact a driving force behind the search for the optimal predictive cycle, hence a primary trigger for the hierarchical construction of metrical structure.

**Figure 8:**
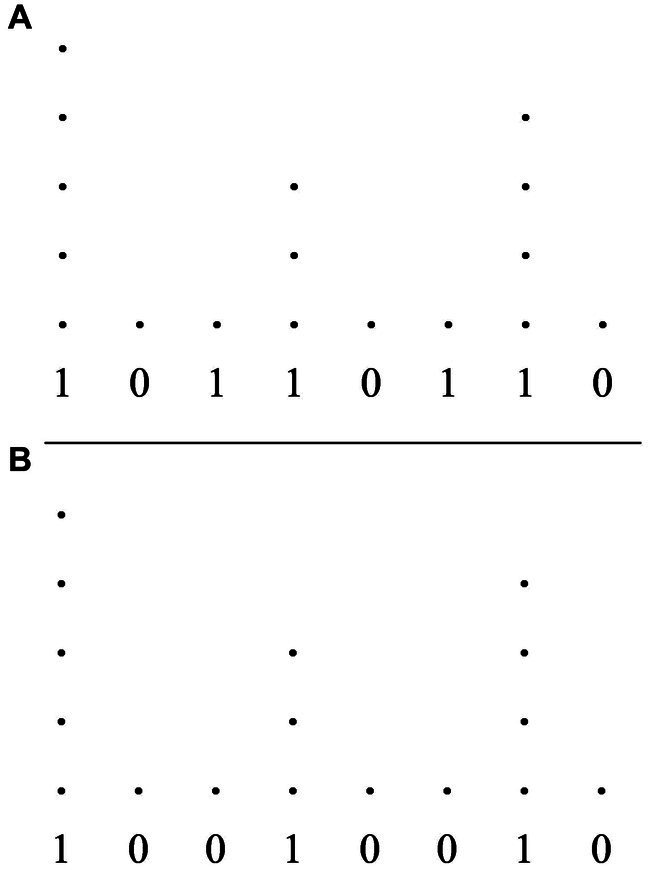
**(A)** Hierarchical predictive structure of the cinquillo rhythm produced by cyclic application, from left to right, of BP to binary subsequences of the rhythm. The dots correspond to nodes in a tree and the position of the dots expresses which label (Strong or Weak) projects. **(B)** Hierarchical predictive structure of the tresillo rhythm produced by cyclic application, from left to right, of BP to binary subsequences of the rhythm. The dots correspond to nodes in a tree and the position of the dots expresses which label (Strong or Weak) projects.

Let us exemplify on the cinquillo (6). Here, a parser in search for the optimal cycle is led from the lower-level prediction that an attack will recur every two pulses within an interval of three pulses, to the higher-level prediction that an attack will recur every three pulses within an interval of eight pulses. There are two noticeable consequences to this processes. First, the parser has established a successive cyclic hierarchy of predictive layers (the cycle of three pulses and the optimal cycle of three pulses). Second, there is now a rather obvious correspondence between the 101 sequence associated to the optimal cycle of the cinquillo and the notion of chunk in language, conceived as a deterministic transition among (atomic) symbols. Crucially, BP applies within a sequence of linearly ordered elements within a chunk. Consider, then, what happens when the parser applies BP to the subsequence of three pulses 101 identified by the IOI associated to the cyclic timeline with a duration of eight pulses. Basically, BP establishes that the linear ordering found within a chunk in terms of precedence must be mirrored by a linear ordering defined in terms of containment. For instance, if 1 precedes 0, then 1 must be contained in 0 or it must contain 0. Now, assume that the pulse sequence 101 in the cinquillo is a sequence of a strong pulse (S) followed by a weak pulse (W) followed by a strong pulse (S). A natural assumption is that BP applies cyclically to binary subsequences. The first binary subsequence in 101 (i.e., 10) is S preceding W. According to BP, if S precedes W, a possibility is that S projects, giving rise to a tree in which S branches into S and W. At this point, the bigram 10 within 101 is represented as 1 (that is, S) and it precedes a second occurrence of 1 (that is, S). Once again, the first S in the sequence projects, giving rise to a second level of projection in which S branches into S-W and S (crucially, the S that projects can be naturally interpreted as metrically more salient than the S that does not project). If we recursively apply this procedure of hierarchy-building within the cycle of eight pulses, we get the representation in Figure 8 (A), where the dots correspond to nodes in a tree and the position of the dots expresses which label (S or W) projects. This representation is roughly equivalent to one of the two metrical structures that we associated to the tresillo in the discussion in Section 4.1 (Figure 6B). In fact, one can easily see that by applying BP to the tresillo, according to the binary procedure illustrated above, we get the metrical structure in Figure 8B, which is the same metrical structure associated with the cinquillo in Figure 8A. We see thus that, with syncopated rhythms, the same hierarchically defined metrical structure is actually compatible with a variety of different rhythms.

As stressed above, the metrical structures in Figure 8 do not represent the unmarked binary meter that is typically associated to syncopated rhythms such as the tresillo and the cinquillo. However, as proposed, this fact is accounted for by the hypothesis that meter does not guide rhythm, but supports cyclic bodily entrainment. As seen above, if we adopt this perspective, we derive the unmarked status of the meter represented in Figure 6A for tresillo from the requirement of isochronous bodily entrainment, which induces a binary division of the cycle, to the effect that for a cycle of eight pulses, we get a division by two, and not the non-isochronous division by three that would result from the hierarchy-building procedure based on BP and is represented in Figure 8.

The conclusions we draw from these observations are that (i) syncopation forces the abandonment of lower-level predictive cycles in favor of higher-level ones; (ii) syncopation dynamically forces the construction of a hierarchy of different predictive layers; (iii) once this hierarchical analysis of rhythm is triggered by syncopation and is in place, actual metrical structure can be bootstrapped by applying BP to the chunks identified through the IOI’s relative to the optimal cycle; (iv) at the same time, the resulting metrical structure may entirely discard the hierarchical process performed by BP in order to produce a model of bodily entrainment that is compatible with relevant constraints of body movement and coordination (for example, in the case of Western music these constraints may be dictated by the need for isochronous bodily entrainment, which is based on the isochronous partition of the optimal cycle).

On these grounds, a number of interesting possibilities emerge concerning the relation between rhythm and language. The most important one – we submit – is that hierarchy should not be presupposed but should be derived, both for language and music. For language, this means that the real issue is not so much how we map rooted directed acyclic graphs into a linear ordering defined in terms of precedence/subsequence but, rather, how we build syntactic trees on the basis of the linear ordering defined by precedence/subsequence. More particularly, what matters for syntax is the arrangement of morphemes, with this arrangement resulting from selection. From this perspective, the relevant question becomes whether there is a way to infer some abstract relation of linear ordering from the relation of selection as defined within the Extended Projection (EP) of lexical categories,^18^ and of modeling the former both as precedence/subsequence and domination (Vender et al., 2023). Interestingly, it can be shown that a procedure of hierarchy-building defined along these lines makes some specific typological predictions, both for the issues related to Greenberg’s Universal 20 (the order of modifiers within noun-phrases; see Delfitto, 2023) and for the issues related to basic sentential order (SVO vs. SOV; see Delfitto and Vender, 2023). These predictions are substantially different from those made by competing approaches to linearization in language, like Kayne’s Antisymmetry (Kayne, 1994).

For rhythm, as we have repeatedly emphasized, this means that it is rhythm that constructs meter, and not the other way around. If we are correct, in both cases structure-building is based on some wired-in bootstrapping condition along the lines of BP.

At the same time, there are reasons not to conflate language and music into the same cluster of neurocognitive factors. Entrainment marks an important difference. If the reasoning above is on the right track, bodily entrainment neutralizes, at least to a certain extent, the potential of hierarchical construction of the metrical space, by imposing, for example, the requirement of isochronous partitions.

Finally, notice that the proposals presented in this contribution potentially address the ultimate question that should be faced in an explanatory theory of language and music: Why should there be a mapping from sequences to hierarchical structures? More explicitly, why should linguistic and musical computations be organized hierarchically rather than linearly? In language, the role of structure is empirically evident but the answer to the question is not trivial (for a recent discussion, see Vender et al., 2023). In the case of rhythm, the parser appears to have a preference for discarding lower-level prediction cycles in favor of higher-level ones, which are computationally more efficient, particularly as models of entrainment. In this respect, we might even speculate that rhythm has been the first and most essential trigger for hierarchy-building in the integrated system of musical and linguistic cognition. Many fascinating questions emerge, which we leave to future research. However, one conclusion is firmly established: If the story we have told is on the right track, syncopation has a large part in it.

## Data availability statement

The original contributions presented in the study are included in the article/supplementary material, further inquiries can be directed to the corresponding author.

## Author contributions

GF: Conceptualization, Formal analysis, Investigation, Resources, Visualization, Writing – original draft, Writing – review & editing. DD: Conceptualization, Formal analysis, Funding acquisition, Supervision, Writing – original draft, Writing – review & editing.

## Footnotes

^1^As correctly pointed out by one of the reviewers, associating the notion of rhythm with that of musical surface does not mean, however, that rhythm should be given an objective, material status. Even though it is true that “rhythm involves the structure of the temporal stimulus” (London, 2012, p. 4), this does not mean that a rhythm should be identified with objective features of the acoustic signal. A rhythm, intended as a sequence of IOI’s, as we do here, is the result of a process of interpretation that has cognitive and, ultimately, neurological underpinnings (Pöppel, 1997) and is also often influenced by the species- and culture-specific strategies of stimuli interpretation (Cameron et al., 2015; Jacoby and McDermott, 2017). On the complex interplay between metrical structure, phenomenal accent, and the listener’s temporal expectations see Parncutt (1994).

^2^London (2012) also adopts a system of well-formedness rules for meter that is notionally similar to that of GTTM but is intended to have broader application than Western Tonal Music. This is achieved by London (2012) by replacing MWFR 3 with his well-formedness condition WFC 5, “Each subcycle must connect nonadjacent time points on the next lowest cycle. For example, each successive segment of the beat cycle must skip over at least one time-point on the N-cycle.” London has convincingly argued that this condition is a universal aspect of metric well-formedness, rather than idiom specific.

^3^The notion of “bootstrapping” has played an important role in linguistics, and most especially in language acquisition, since Roger Brown’s work in the 1950’s. Of particular relevance in the context of the current discussion is the mechanism of “phonological bootstrapping” (Morgan and Demuth, 1996) hypothesized to explain how infant may start acquiring the lexicon and syntax of their native language from a purely phonological analysis of the speech signal.

^4^See Delfitto (2023) for an extension of BP to the full architecture of syntax, including the phrases (specifiers) selected by the elements belonging to the UEP, which encode adverbial modification besides argument and event structure.

^5^The work of Witek et al. (2014) capitalizes on previous research concerning the link between pleasure and emotional responses to music, on the one hand, and expectation and anticipation, on the other. These include Gebauer et al. (2012), Vuust and Kringelbach (2010), Vuust and Frith (2008), Huron (2006), Meyer (1956), and Gomez and Danuser (2007). The importance of syncopation *per se* in groove has recently been questioned by Sioros et al. (2022). It is also important to keep in mind that there are other important predictors of groove besides syncopation, including familiarity and taste (Senn et al., 2019).

^6^Berlyne (1971) famously proposed an inverted U-shaped curve (or Wundt curve) reflecting a general relationship between appreciation and structural complexity in art. Empirical support for Berlyne’s proposal in the domain of music was first produced by Heyduk (1975). The inverted U-shape relation between groove and syncopation has been challenged by Sioros et al. (2022) who investigated the effects of syncopation in polyphonic music. The participants in the study were asked to evaluate their desire to move to the music excerpts taken from funk and rock songs and to variations of these excerpts generated algorithmically, some of them de-syncopated and some of them with an increased amount of syncopation. Sioros et al. (2022, p. 503) report that “While the original excerpts were rated higher than the de-syncopated, the algorithmic syncopation was not as successful in evoking groove. Consequently, a moderate level of syncopation increases groove, but only for certain syncopation patterns.” As the current contribution focuses on monophonic rhythms, we leave a full discussion of syncopation in polyphonic contexts to a different occasion.

^7^See also Sioros et al. (2018).

^8^See also Sioros et al. (2022).

^9^See also Sioros (2023).

^10^Suggested by a reviewer.

^11^For a discussion of the mechanisms guiding the detection of isochronous patterns in human perception and their evolutionary underpinnings, see Ravignani and Madison (2017).

^12^More concretely, we can envision this strategy being implemented within a model such as Dynamic Predictive Coding (Jiang and Rao, 2022), which is a hierarchical model of spatiotemporal prediction and sequence learning that “assumes that higher cortical levels modulate the temporal dynamics of lower levels, correcting their predictions of dynamics using prediction errors” (p. 1) and in which “as a result, lower levels form representations that encode sequences *at shorter timescales* (e.g., a single step) while higher levels form representations that encode sequences at *longer timescales* (e.g., an entire sequence)” (p. 1; italics are ours). The model is supported by experimental evidence in the visual domain but, importantly, the authors envision an extension of the model to “action-conditioned prediction and hierarchical planning” (p. 11).

^13^Of course, this is not to suggest that isochronicity is a necessary requirement of all meters. Different musical idioms may, in fact, adopt different preferences in modelling their bodily entrainment to music.

^14^The bottom-up approach proposed also resonates with a school of thought in the philosophy of music, most prominently represented by Christopher Hasty, that purports to return value to rhythm as an actual, incarnated experience of making music – “a process of event-formation in which repetition is transformed into novelty *and felt as such*” (Hasty, 2019, p. 235) as opposed to an abstract representation of it. Indeed, there is a similarity between the bootstrapping strategy described in the previous section and the mechanism of “projection” proposed by Hasty (1997). However, it is important to observe that, whereas the mechanism of projection was meant by Hasty as an alternative to the notion of meter, the bootstrapping procedure at the core of our hypothesis is a mechanism that informs meter but does not replace it.

^15^The proposed bottom-up perspective raises the issue of how to characterize the perspective of the performer, as opposed to that of the listener. This issue would require a detailed discussion of the available evidence on the topic, which, for reason of space, we defer to a different occasion. For the time being, we only point out that the framework proposed does entail that rhythm elicits bodily entrainment by providing a model of temporal cyclicities but does not entail that rhythm is the only way to entertain in cognition a model of bodily entrainment. In a nutshell, the goal of the performer is that of entertaining a model of bodily entrainment and “communicate” it to the listener by using rhythm as a means of transmission. Rhythm becomes, from this perspective, a means of transmission/communication of movement.

^16^The co-operation or competition of different bottom-up strategies could be modeled within an Optimality Theory model (Prince and Smolensky, 2004) which, interestingly, has been proposed as a model for both language and music (Gilbers and Schreuder, 2002, Language and music in Optimality Theory. From Rutgers Optimality Archive (http://roa.rutgers.edu), ROA # 517-0103).

^17^We thank one of the reviewers for suggesting this case-study.

^18^By Extended Projection (EP) we mean the syntactic representation projected from the lexicon that observes the subcategorization properties of lexical items (Chomsky, 1981, p. 29).
